# PyRhO: A Multiscale Optogenetics Simulation Platform

**DOI:** 10.3389/fninf.2016.00008

**Published:** 2016-03-11

**Authors:** Benjamin D. Evans, Sarah Jarvis, Simon R. Schultz, Konstantin Nikolic

**Affiliations:** ^1^Centre for Bio-Inspired Technology, Institute of Biomedical Engineering, Department of Electrical and Electronic Engineering, Imperial College LondonLondon, UK; ^2^Centre for Neurotechnology, Institute of Biomedical Engineering, Department of Bioengineering, Imperial College LondonLondon, UK

**Keywords:** optogenetics, opsin, Python, Jupyter, PyRhO, spiking neurons, NEURON simulator, Brian simulator

## Abstract

Optogenetics has become a key tool for understanding the function of neural circuits and controlling their behavior. An array of directly light driven opsins have been genetically isolated from several families of organisms, with a wide range of temporal and spectral properties. In order to characterize, understand and apply these opsins, we present an integrated suite of open-source, multi-scale computational tools called PyRhO. The purpose of developing PyRhO is three-fold: (i) to characterize new (and existing) opsins by automatically fitting a minimal set of experimental data to three-, four-, or six-state kinetic models, (ii) to simulate these models at the channel, neuron and network levels, and (iii) provide functional insights through model selection and virtual experiments *in silico*. The module is written in Python with an additional IPython/Jupyter notebook based GUI, allowing models to be fit, simulations to be run and results to be shared through simply interacting with a webpage. The seamless integration of model fitting algorithms with simulation environments (including NEURON and Brian2) for these virtual opsins will enable neuroscientists to gain a comprehensive understanding of their behavior and rapidly identify the most suitable variant for application in a particular biological system. This process may thereby guide not only experimental design and opsin choice but also alterations of the opsin genetic code in a neuro-engineering feed-back loop. In this way, we expect PyRhO will help to significantly advance optogenetics as a tool for transforming biological sciences.

## 1. Introduction

Optogenetics is a biotechnology which renders excitable cells light-sensitive by inserting genes which, upon expression, create light-activated ion channels known originally as rhodopsins (Nagel et al., 2003; Boyden et al., 2005). Over the last 10 years optogenetics has found widespread application, initially in neuroscience (Zhang et al., 2006; Adamantidis et al., 2007; Arenkiel et al., 2007; Han and Boyden, 2007; Yizhar et al., 2011), but increasingly also in more distal areas of physiology such as cardiac science (Arrenberg et al., 2010; Boyle et al., 2013), intracellular signaling (Airan et al., 2009), and gene transcription (Konermann et al., 2013). Applications to date have included control of motor cortex (Aravanis et al., 2007), cortical circuit mapping (Wang et al., 2007; Zhang et al., 2007; Ayling et al., 2009; Petreanu et al., 2009; Klapoetke et al., 2014), optoelectronic neuroprosthetic devices (for example retinal Lagali et al., 2008; Degenaar et al., 2009; Busskamp and Roska, 2011 or cochlear prostheses, Hernandez et al., 2014), regulation of the symptoms of neurodegenerative disorders (e.g., Parkinson's Gradinaru et al., 2009), closed loop control of epileptic seizures, peripheral nerve stimulation (Arlow et al., 2013), and novel cardiac pacemaker technology (Bruegmann and Sasse, 2015), to name just a few.

In an effort to develop more effective and tailored opsins, hybrids and genetic mutants are continually being created (Berndt et al., 2008; Lin et al., 2009; Hegemann and Moglich, 2011; AzimiHashemi et al., 2014). Experimentally characterizing these new variants is a lengthy process requiring substantial effort before they can be harnessed to address questions in neuroscience (Chow et al., 2010; Gunaydin et al., 2010; Lin, 2011; Chuong et al., 2014). The problem is further compounded when considering the number of combinations between opsins (with total variations now in the hundreds, Zhang et al., 2011) and target cell types. Experimentally testing each combination of opsin and target cell type of interest is practically impossible, effectively limiting the use of optogenetics as a tool.

Theoretical understanding of the underlying mechanisms of optogenetics has developed over the past 10 years (Hegemann et al., 2005; Feldbauer et al., 2009), which has led to a deeper understanding of the biophysical mechanisms of the photosensitization agents which form the foundations of optogenetics (Hegemann et al., 2005; Bamann et al., 2008; Ernst et al., 2008; Nikolic et al., 2009; Stehfest and Hegemann, 2010). Furthermore, the design and engineering of optogenetic devices must start with models of the underlying molecular mechanisms of opsin behavior in cells (Gradinaru et al., 2007; Nikolic et al., 2007; Shoham and Deisseroth, 2010; Foutz et al., 2012; Williams et al., 2013). Computational modeling is thus core to understanding how light induced ionic transport across cell membranes can be tailored for different applications: from probing cellular physiology to creating new treatments for neurological and psychiatric illnesses.

The quest to both expand and refine optogenetics as an effective tool for neuroscience and other areas of physiology requires multiple levels of analysis: from molecular modeling through kinetic models and even network level models. To aid in this effort we propose *PyRhO*; an integrated suite of open-source, multi-scale computational tools to characterize opsins, then rapidly develop and conduct virtual experiments with them *in silico*.

PyRhO offers several integrated computational tools for analysing and experimenting with (rhod)opsins in a virtual environment:

1. The first tool will automatically fit a choice of models to experimental data, extracting the parameters that describe the functional dynamics of the opsins.
2. The second tool can then take these extracted parameters (or alternatively use default values) and simulate a wide range of experimental protocols to reproduce the photo-response of the opsin of interest. These protocols are typically voltage-clamp experiments and include common engineering inputs such as steps, ramps, and chirps, along with more tailored protocols such as pairs of increasingly spaced pulses for evaluating the recovery process.
3. These models and protocols can be run on several simulation platforms spanning multiple scales (to model isolated opsins or transfected neurons) including:
  - Pure Python for simple channel-level voltage clamp experiments;
  - NEURON for morphologically detailed models of optogenetically transfected neurons;
  - Brian2 for simulating whole networks with transfected groups of neurons.
4. A Graphical User Interface (GUI) for easy navigation through all tools, running of virtual experiments and sharing of results.

In this way, PyRhO allows the investigator to simulate opsin dynamics on multiple scales from sub-cellular channels, to individual neurons and finally the dynamics of whole networks. This will help to elucidate the link between the biophysics of opsins and the functional implications of their use in a particular biological system.

The tools are written in Python due to its rapidly growing popularity across the sciences, readability, modularity and large array of open-source modules (Muller et al., 2015). An accompanying GUI running in IPython/Jupyter (Pérez and Granger, 2007) has also been developed to facilitate more interactive exploration of the models for both experimental and pedagogic purposes, requiring virtually no programming experience. In addition to controlling the fitting routines, the GUI also exposes the integrated simulators (e.g., NEURON). Furthermore, this self-logging, notebook-based approach has been identified as a particularly promising medium for sharing models and reproducing results in computational neuroscience (Topalidou et al., 2015).

Simulations based on these virtual opsins will enable neuroscientists to gain insight into their behavior and rapidly identify the most suitable variant for application in a particular biological system, not only guiding choice, but also opsin development. Understanding gained from biologically realistic simulations may provide ideas of how to alter the opsin's genetic code to generate new mutants. These new variations can then be characterized and simulated within PyRhO to determine their suitability for a particular application.

Here, we describe the structure of PyRhO and demonstrate a sample of its capabilities, illustrated through snippets of code and its GUI. We demonstrate the use of PyRhO in fitting models to Channelrhodopsin-2 (ChR2) data and present results for typical illumination strategies and experimental protocols designed to tease apart the effects of key model parameters. We finish with a discussion of the main benefits of using PyRhO, its limitations to date and planned future developments to extend its capabilities.

## 2. Materials and methods

PyRhO is written as a Python module and released as an open-source project under the revised BSD license. Download and installation instructions can be found with the code at PyRhO's GitHub repository (https://github.com/ProjectPyRhO/PyRhO), along with example notebooks and a link to the project's website containing further information. A virtual machine with all dependencies installed and examples ready to run is also available, such that the GUI can be used with a minimum of set-up and virtually no programming experience.

The module is comprised of several integrated components for fitting model parameters to experimental data and for simulating the models at multiple scales. Fitting data is an optional step since PyRhO is initialized with default parameters, allowing the user to immediately experiment with simulating the three types of opsin models in order to better understand their dynamics. If the required data are provided to the fitting algorithms however, the parameterized models may be run through the stimulation protocols to efficiently characterize the opsins *in silico*, or determine their suitability for a particular application based upon their dynamics.

### 2.1. Implementation

PyRhO is implemented as a Python package called pyrho which builds upon popular scientific Python modules including scipy, numpy, matplotlib, and lmfit. Additionally, if optogenetic simulations in detailed morphological models of individual (or a few) neurons are required, NMODL files (Hines and Carnevale, 2000) are provided for use with NEURON (Hines et al., 2009). Similarly, for network-level simulations PyRhO has been integrated with the Brian simulator (Goodman and Brette, 2008, 2009) and includes model descriptions suitable for use with Brian2.

**Figure 1:**
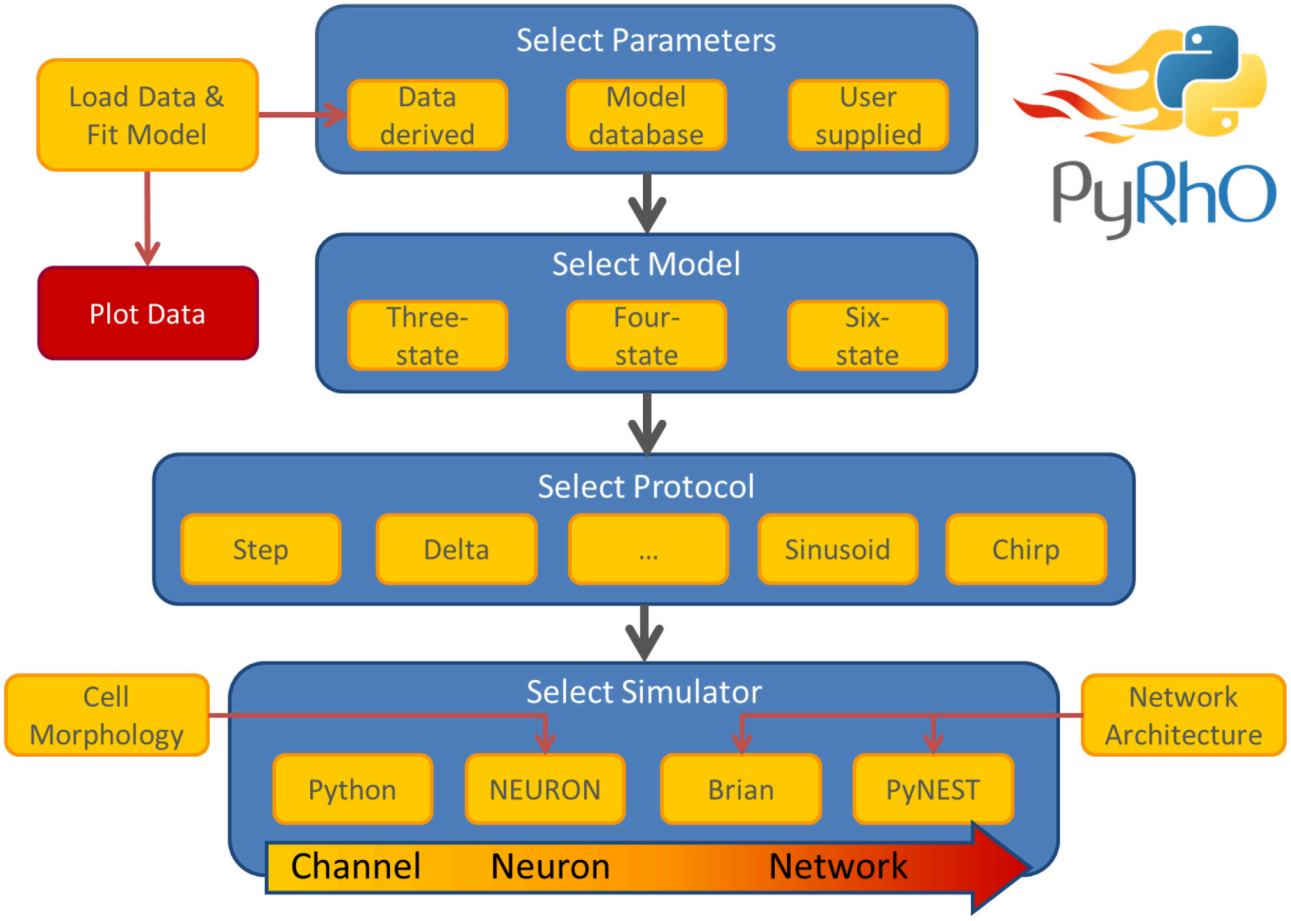
**Schematic of the PyRhO work-flow**. Model parameters may be user supplied, initialized with defaults, or optionally derived from data. The user then selects the number of states in the model, the stimulation protocol and the simulation engine to start running virtual experiments.

The simulation architecture is designed around three layers of abstraction: models, protocols and simulators. These layers are illustrated in the work-flow schematic of Figure 1 along with the other major components of PyRhO. Each layer contains families of classes to create a uniform interface for each subclass, for example, the differences in setting the light-dependent transition rates of the three models are shielded from the user by endowing each opsin model subclass with the method setLight(). A similar approach is taken with the other layers providing a common set of member variables and methods, making usage consistent and providing a framework for future development of new subclasses (i.e., additional kinetic models, stimulation protocols, and simulation platforms).

### 2.2. Photocurrent model

A detailed understanding of how the channel is gated and the ions are conducted is still lacking, although some recent studies have elucidated important aspects of the pore formation and ionic transport (Feldbauer et al., 2009; Kuhne et al., 2014) We assume that all light-sensitive ion channel currents (*I*) can be expressed in the classic form:

(1) $$\begin{array}{l} I=g\cdot \left(v-E\right)\;, \end{array}$$

where *g* is the channel conductance, *v* the membrane voltage and *E* is the reversal potential for the specific opsin type. Generally speaking the ionic conductance is a complex function of light flux (ϕ(*t*)), wavelength (λ), and the opsin's photocycle, membrane voltage, temperature (*T*), and intracellular and extracellular pH (Gradmann et al., 2011). We use a simplified empirical form for the channel conductance, introduced by Hodgkin and Huxley, expressing it as a product of a constant (*g*_0_, in our case this is maximum conductance at *v* = −70*mV*), and a numerical coefficient (*f* > 0):

(2) $$\begin{array}{l} g=g\left(\phi ,\lambda ,v,T,pH,t\right)=g_{0}\cdot f\left(\phi ,\lambda ,v,T,pH,t\right)\;, \end{array}$$

In this version of PyRhO we have implemented the photocycle and membrane voltage dependencies and assumed that these two contributions can be separated:

(3) $$\begin{array}{l} g=g_{0}\cdot f_{\text{ϕ}}\left(\phi ,t\right)\cdot f_{\text{v}}\left(v\right)\;. \end{array}$$

These two dependences are considered to be the most relevant for physiological electrolyte conditions, when temperature and pH are considered to be fixed. Other dependencies will be implemented in the next version of PyRhO.

### 2.3. Photocycle models

**Figure 2:**
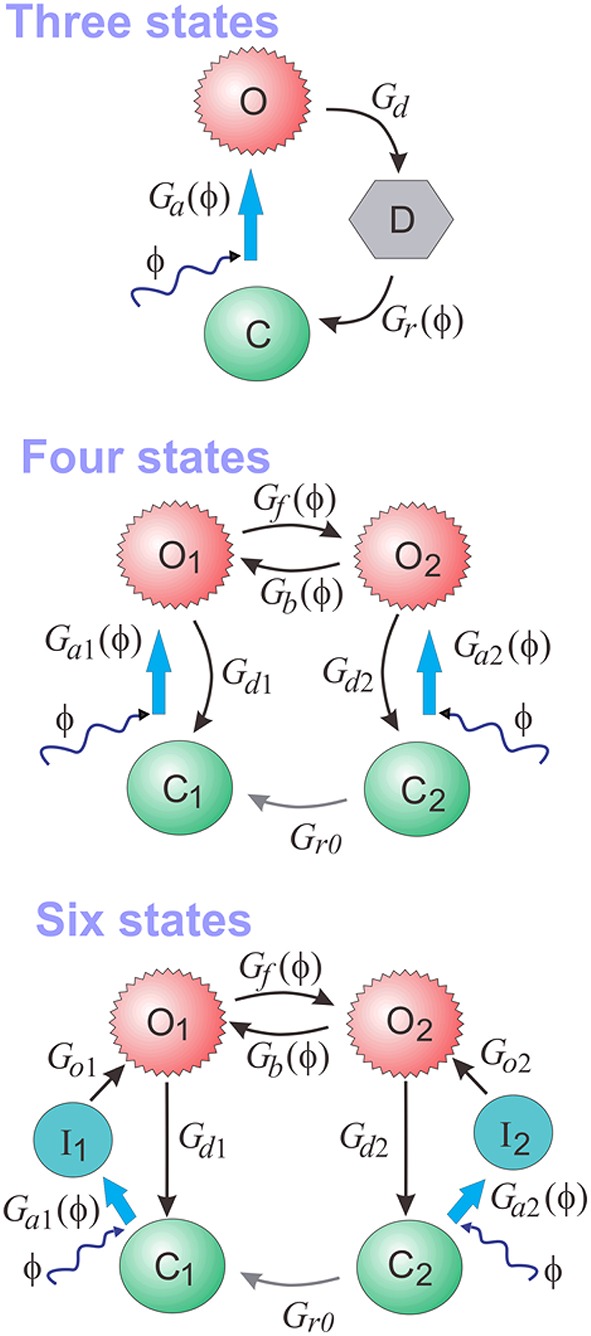
**The three-, four-, and six-state functional Markov models of opsins**.

At the core of PyRhO are three functional Markov models of opsin kinetics, namely the three-, four- (Nikolic et al., 2009), and six-state (Grossman et al., 2013) models. We note that very similar models have been investigated in several other studies (Gradmann et al., 2002; Nagel et al., 2003; Hegemann et al., 2005; Ishizuka et al., 2006; Bamann et al., 2008; Ernst et al., 2008; Foutz et al., 2012; Williams et al., 2013) but used our earlier models as a starting point as we have since extended them and unified their notation. These models vary in complexity providing a range in the trade-off between biological accuracy and computational efficiency to choose from. The key features of these models, including an outline of their strengths and weaknesses, are summarized in Table 1 with accompanying illustrations in Figure 2. Since their original formulation, the models have been extended to encompass additional parameter dependencies, better fit the experimental data and use a consistent notation, with the full model descriptions given in Table 2. An analytic solution for the three-state model was also calculated and is included in the Appendix.

**Table 1:** **Summary of opsin models**.

**States**	**Transitions**	**Parameters**	**Pros**	**Cons**
3	3	11	Efficient analytic solution	Single exponential off-phase decay
4	7	17	Balance of detail and efficiency	Lacks short-pulse dynamics
6	9	19	Most detailed dynamics	Computationally expensive

**Table 2:** **Opsin model equations**.

**States**	**Functional state equations**	**Light-dependent transitions**	**Conductance factors**
3	$\begin{array}{ll} Ċ & =G_{r}\left(\phi \right)D-G_{a}\left(\phi \right)C \end{array}$ $\begin{array}{ll} Ȯ & =G_{a}\left(\phi \right)C-G_{d}O \end{array}$ $\begin{array}{ll} Ḋ & =G_{d}O-G_{r}\left(\phi \right)D \end{array}$ *C* + *O* + *D* = 1	$\begin{array}{ll} G_{a}\left(\phi \right) & =k_{a}\frac{\phi^{p}}{\phi^{p}+\phi_{m}^{p}} \end{array}$ $\begin{array}{ll} G_{r}\left(\phi \right) & =k_{r}\frac{\phi^{q}}{\phi^{q}+\phi_{m}^{q}}+G_{r0} \end{array}$	$\begin{array}{ll} f_{\text{ϕ}}\left(\phi \right) & =O \end{array}$ $\begin{array}{ll} f_{\text{v}}\left(v\right) & =\frac{1-e^{-\left(v-E\right)∕v_{0}}}{\left(v-E\right)∕v_{1}} \end{array}$
4	$\begin{array}{ll} \dot{C_{1}} & =G_{d1}O_{1}+G_{r0}C_{2}-G_{a1}\left(\phi \right)C_{1} \end{array}$ $\begin{array}{ll} \dot{O_{1}} & =G_{a1}\left(\phi \right)C_{1}+G_{b}\left(\phi \right)O_{2}-\left(G_{d1}+G_{f}\left(\phi \right)\right)O_{1} \end{array}$ $\begin{array}{ll} \dot{O_{2}} & =G_{a2}\left(\phi \right)C_{2}+G_{f}\left(\phi \right)O_{1}-\left(G_{d2}+G_{b}\left(\phi \right)\right)O_{2} \end{array}$ $\begin{array}{ll} \dot{C_{2}} & =G_{d2}O_{2}-\left(G_{r0}+G_{a2}\left(\phi \right)\right)C_{2} \end{array}$ *C*_1_ + *O*_1_ + *O*_2_ + *C*_2_ = 1	$\begin{array}{ll} G_{a1}\left(\phi \right) & =k_{1}\frac{\phi^{p}}{\phi^{p}+\phi_{m}^{p}} \end{array}$ $\begin{array}{ll} G_{f}\left(\phi \right) & =k_{f}\frac{\phi^{q}}{\phi^{q}+\phi_{m}^{q}}+G_{f0} \end{array}$ $\begin{array}{ll} G_{b}\left(\phi \right) & =k_{b}\frac{\phi^{q}}{\phi^{q}+\phi_{m}^{q}}+G_{b0} \end{array}$ $\begin{array}{ll} G_{a2}\left(\phi \right) & =k_{2}\frac{\phi^{p}}{\phi^{p}+\phi_{m}^{p}} \end{array}$	$\begin{array}{ll} f_{\text{ϕ}}\left(\phi \right) & =O_{1}+\gamma O_{2} \end{array}$ $\begin{array}{ll} f_{\text{v}}\left(v\right) & =\frac{1-e^{-\left(v-E\right)∕v_{0}}}{\left(v-E\right)∕v_{1}} \end{array}$
6	$\begin{array}{ll} \dot{C_{1}} & =G_{d1}O_{1}+G_{r0}C_{2}-G_{a1}\left(\phi \right)C_{1} \end{array}$ $\begin{array}{ll} \dot{I_{1}} & =G_{a1}\left(\phi \right)C_{1}-G_{o1}I_{1} \end{array}$ $\begin{array}{ll} \dot{O_{1}} & =G_{o1}I_{1}+G_{b}\left(\phi \right)O_{2}-\left(G_{d1}+G_{f}\left(\phi \right)\right)O_{1} \end{array}$ $\begin{array}{ll} \dot{O_{2}} & =G_{o2}I_{2}+G_{f}\left(\phi \right)O_{1}-\left(G_{d2}+G_{b}\left(\phi \right)\right)O_{2} \end{array}$ $\begin{array}{ll} \dot{I_{2}} & =G_{a2}\left(\phi \right)C_{2}-G_{o2}I_{2} \end{array}$ $\begin{array}{ll} \dot{C_{2}} & =G_{d2}O_{2}-\left(G_{r0}+G_{a2}\left(\phi \right)\right)C_{2} \end{array}$ *C*_1_ + *I*_1_ + *O*_1_ + *O*_2_ + *I*_2_ + *C*_2_ = 1	$\begin{array}{ll} G_{a1}\left(\phi \right) & =k_{1}\frac{\phi^{p}}{\phi^{p}+\phi_{m}^{p}} \end{array}$ $\begin{array}{ll} G_{f}\left(\phi \right) & =k_{f}\frac{\phi^{q}}{\phi^{q}+\phi_{m}^{q}}+G_{f0} \end{array}$ $\begin{array}{ll} G_{b}\left(\phi \right) & =k_{b}\frac{\phi^{q}}{\phi^{q}+\phi_{m}^{q}}+G_{b0} \end{array}$ $\begin{array}{ll} G_{a2}\left(\phi \right) & =k_{2}\frac{\phi^{p}}{\phi^{p}+\phi_{m}^{p}} \end{array}$	$\begin{array}{ll} f_{\text{ϕ}}\left(\phi \right) & =O_{1}+\gamma O_{2} \end{array}$ $\begin{array}{ll} f_{\text{v}}\left(v\right) & =\frac{1-e^{-\left(v-E\right)∕v_{0}}}{\left(v-E\right)∕v_{1}} \end{array}$

Both four- and six-state models assume that there are two open states (*O*_1_ and *O*_2_, see Figure 2), with channel conductances *g*_O_1__ and *g*_O_2__, respectively. The Photocycle factor in Equation (1) has the form:

(4) $$\begin{array}{l} f_{\text{ϕ}}\left(\phi \right)=O_{1}+\gamma O_{2}\;, \end{array}$$

where *O*_1_ and *O*_2_ are the fractions of opsins in two open states in the interval [0, 1], and γ = *g*_O_2__ ∕ *g*_O_1__. In contrast, the three-state model assumes only one open state (*O*) making the photocycle factor simply: *f*_ϕ_(ϕ) = *O*.

### 2.4. Voltage dependence

Here, we assume that the membrane voltage affects only the ion-channel conductance but not the channel kinetics. By investigating experimental results for Channelrhodopsin-2 steady-state current vs. clamped voltage, the *I*–*V* curve shows inwardly rectifying behavior (Bamberg et al., 2008). We have previously found that an exponential function gives a good fit for this dependency (Grossman et al., 2011), therefore for the voltage factor in Equation (1) we adopt the form:

(5) $$\begin{array}{l} f_{\text{v}}\left(v\right)=\frac{v_{1}}{v-E}\cdot \left(1-e^{-\frac{v-E}{v_{0}}}\right)\;, \end{array}$$

where *v*_0_ and *v*_1_ are fitting parameters, along with *E*, the channel's reversal potential. The exponential dependence on *v* transforms into a linear dependence for large values of *v*_0_ which cause the exponent to be small and the expression in Equation (5) reduces to *f*_v_(*v*) ≈ *v*_1_ ∕ *v*_0_ = *const*, i.e., no direct dependence on membrane voltage, which may be a more appropriate form for some opsins. The expression given by Equation (5) therefore generalizes to both cases for appropriate choices of the parameters *v*_0_ and *v*_1_.

Furthermore, since the voltage dependence factor is defined to be equal to 1 at −70 mV (*f*_v_(−70 mV): = 1), the value of v_1_ is related to the other parameters through the following equation:

(6) $$\begin{array}{l} v_{1}=\frac{70+E}{e^{\frac{70+E}{v_{0}}}-1} \end{array}$$

This relationship is used as a constraint in the fitting procedures described below.

### 2.5. Model fitting

PyRhO incorporates novel fitting algorithms with which each of the opsin models may be parameterized when given an appropriate set of data. The fitting algorithms use the lmfit module (Newville et al., 2014) which in addition to providing access to a variety of optimization algorithms, enables numerical bounds to be placed on parameter values as well as algebraic constraints. Parameters may also be manually fixed if, for example, they have been directly measured experimentally. Once the algorithm has finished, a Parameters object is returned, populated with the extracted parameter values which may then be saved or used as the basis for simulations. Plots of photocurrents simulated with these derived parameters are drawn over the experimental data (with residual error) to allow for visual comparison of the resultant fits.

#### 2.5.1. Characterization data

In order to characterize each model, a set of voltage-clamped photocurrents are required, ideally collected from HEK cells to eliminate the confounding effects of other ion channels which may be present in neurons. To capture all currently modeled variable dependencies, data from three stimulation protocols are necessary, listed below by the model properties which they reveal. In the event of scarce data or uncharacterized variables which the user does not intend to vary, we describe the minimum set of data for the fitting procedure below and discuss the implications for the resultant model.

**Figure 3:**
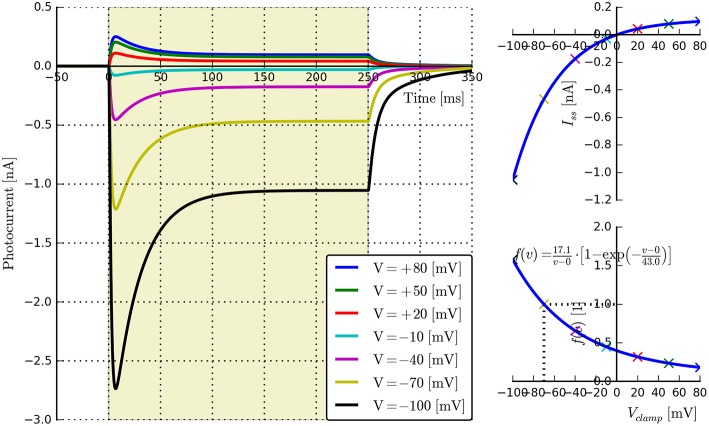
**Photocurrent plots from the “rectifier” protocol with accompanying steady-state current and fitted *f*(*v*) plots**.

**Figure 4:**
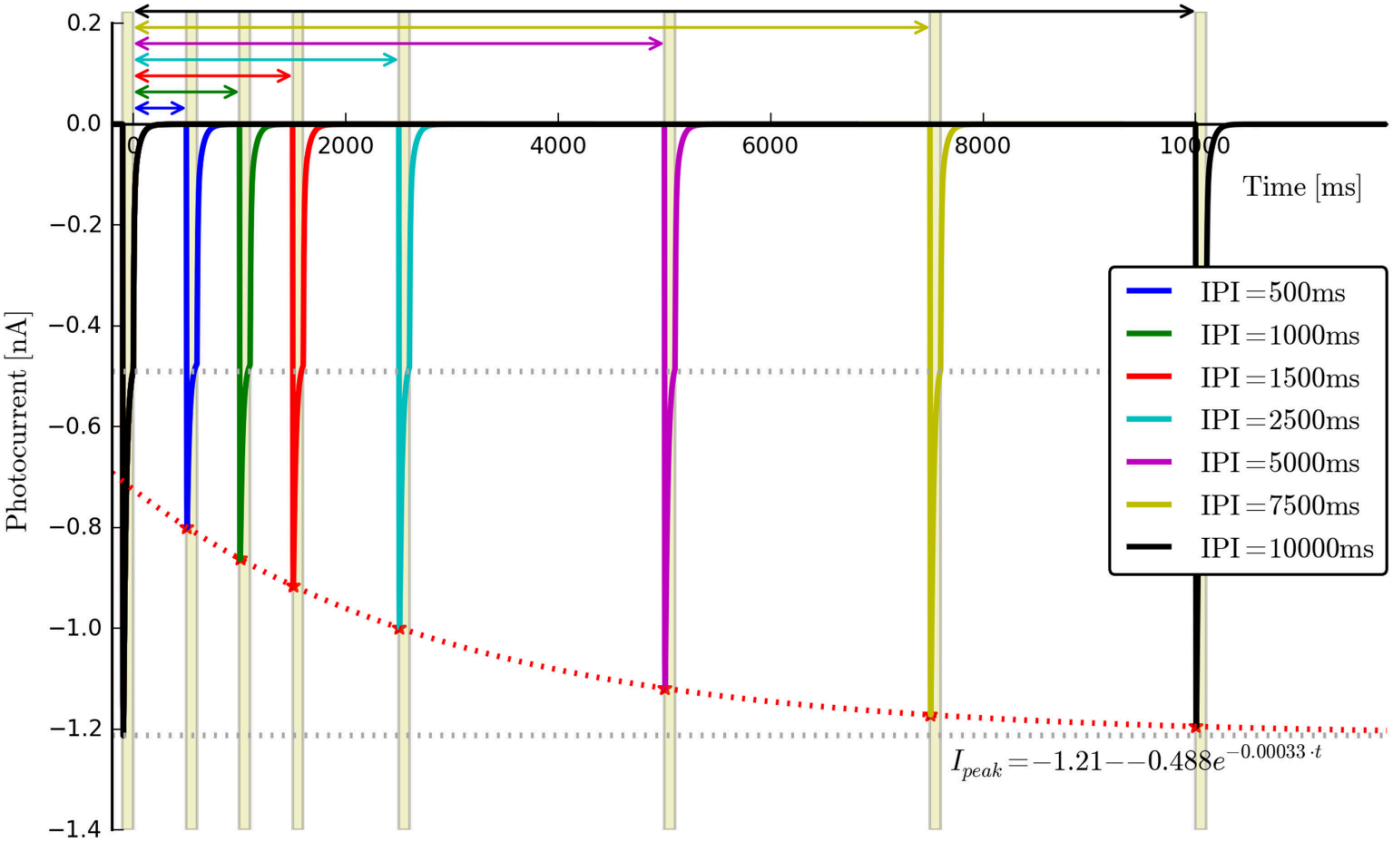
**Photocurrent plots from the “recovery” protocol with fitted peak recovery function**.

**Figure 5:**
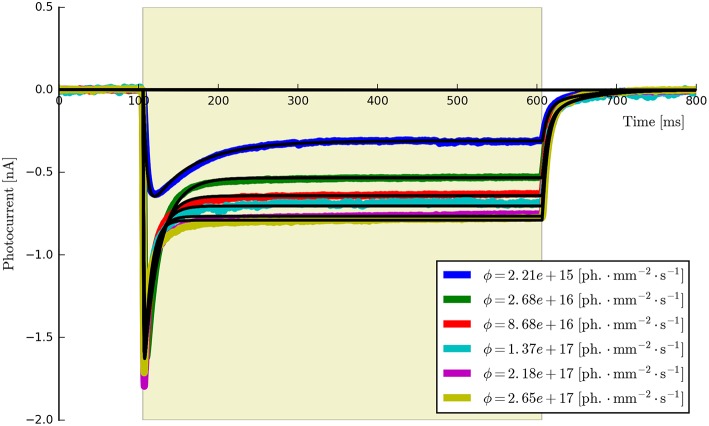
**The six-state model fit to a set of six ChR2 photocurrents using the same model parameters**.

- **Voltage dependence**: {*E, v*_0_, *v*_1_}. Long light pulse (fixed flux) to steady-state, vary voltage clamp potential (e.g., in steps of 30 mV: *V*_clamp_ = {−100, −70, −40, −10, 20, 50, 80} mV, *n* ≥ 5). Voltage clamp values should not be too close to *E* as this may cause distortions in the fitting algorithms. The software will automatically find the plateau values *I*_ss_, plot *I*_ss_ vs. *V*_clamp_, and find the fitting parameters for the function *f*_v_(*v*) given by Equation (5). An example is shown in Figure 3.
- **Recovery rate**: {*G*_*r*0_}. Two long light pulses with varying inter-pulse-interval (IPI), Voltage clamp: −70 *mV*. Light on (first pulse)—light off for e.g., *t*_IPI_ = {0.5, 1, 2.5, 5, 10}s—light on (second pulse). The software will automatically find the peak values for each recording, align the data to the end of the first pulse and fit an appropriate exponential curve of the form $I_{\text{peak}}\left(t\right)=I_{peak0}-a\cdot e^{-G_{r0}\cdot t_{\text{IPI}}}$. We note here that this expression is strictly speaking correct only when both *O*_1_ and *O*_2_ states are empty. Consequently *a* is left as a free fitting parameter and very short values for *t*_IPI_ should be avoided to prevent the distortions caused by the faster transitions. An example for wild-type ChR2 is given in Figure 4, where *t*_IPI_ ≳ 100 ms.
- **Flux dependence**: *Off-curve*: {*G*_*d*(1, 2)_, [*G*_*f*0_, *G*_*b*0_]}; *On-curve*: {All other parameters}. Voltage clamp (preferably): −70 mV, long pulse to steady-state, (e.g., *T* ≈ 500 ms) plus decay of off-curve. Vary light intensity from near threshold to saturation (e.g., ϕ = {0.1, 0.5, 1, 5, 10, 50, 100} mW∕mm^2^, *n* ≥ 5). The recorded off- and on-curves are automatically fitted. An example set is shown in Figure 5 with more details of the algorithm given in Appendix Section (Model-Dependent Fitting Procedures).

Additionally the six-state model requires one or more very short pulses in order to characterize the opsin activation rates which model the lag in transitioning to conductive states upon light stimulation:

- **Opsin activation rate**: {*G*_*o*1_, *G*_*o*2_} One or more *short* pulses, voltage clamp: −70 mV. Vary pulse length, e.g., 0.5, 1, 2, 3, varied up to 10 ms. PyRhO will automatically find the time of the peak current and use an iterative formula to estimate *G*_*o*1_. We initially assume *G*_*o*2_ = *G*_*o*1_. Further details of the algorithm are given in Appendix section Six-state opsin activation rate fitting (Step 1b.).

**Figure 6:**
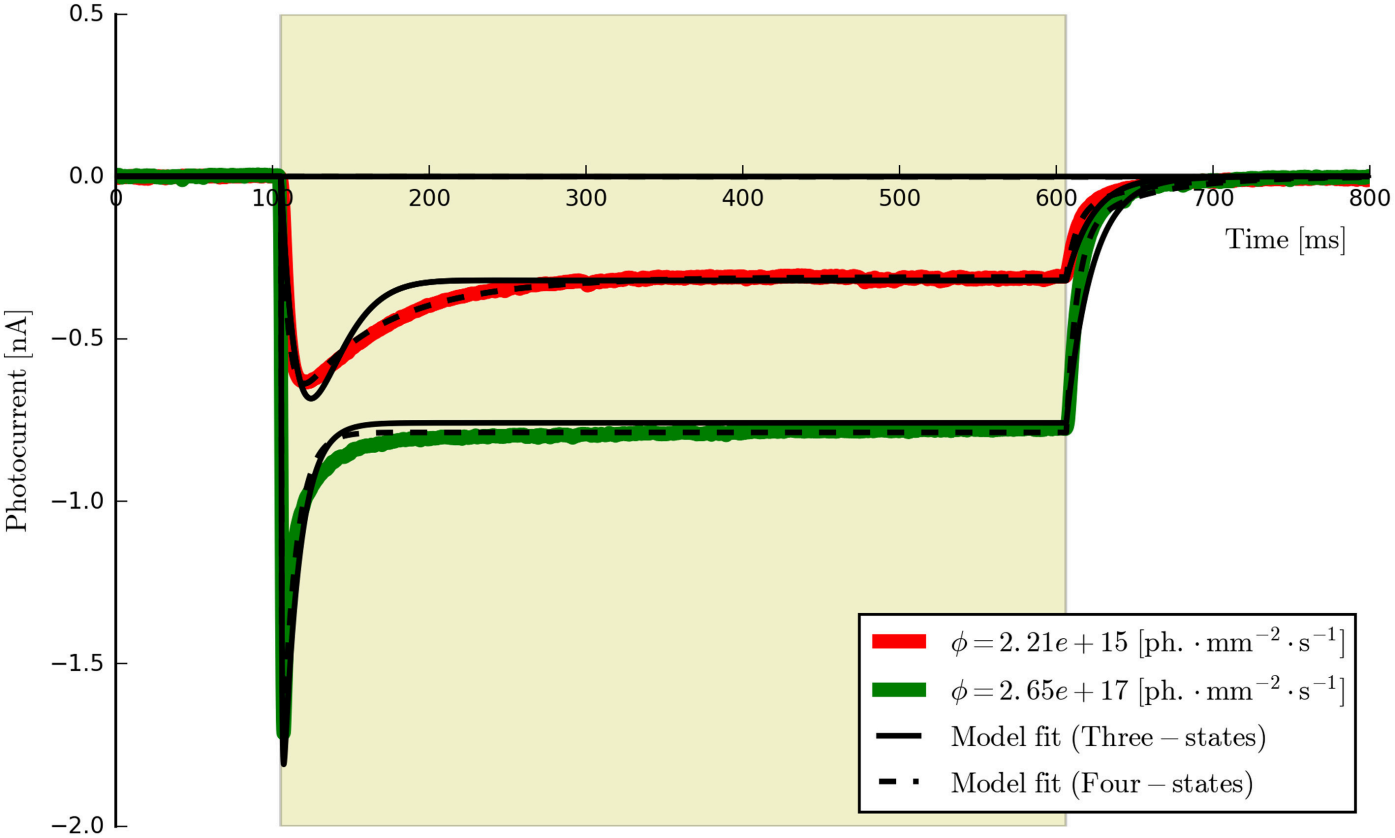
**The three- and four-state models fitted to the first two ChR2 photocurrents for comparison (the six-state fits are omitted since they are almost indistinguishable from the four-state fits at this time scale)**. While both models capture the major features of the data, the four-state (and six-state) models produce a higher quality of fit, particularly during the post-peak inactivation phase (where the three-state curves tend to over-shoot the data) and the deactivation (off) phases which are better described by a double rather than a single exponential decay process.

**Figure 7:**
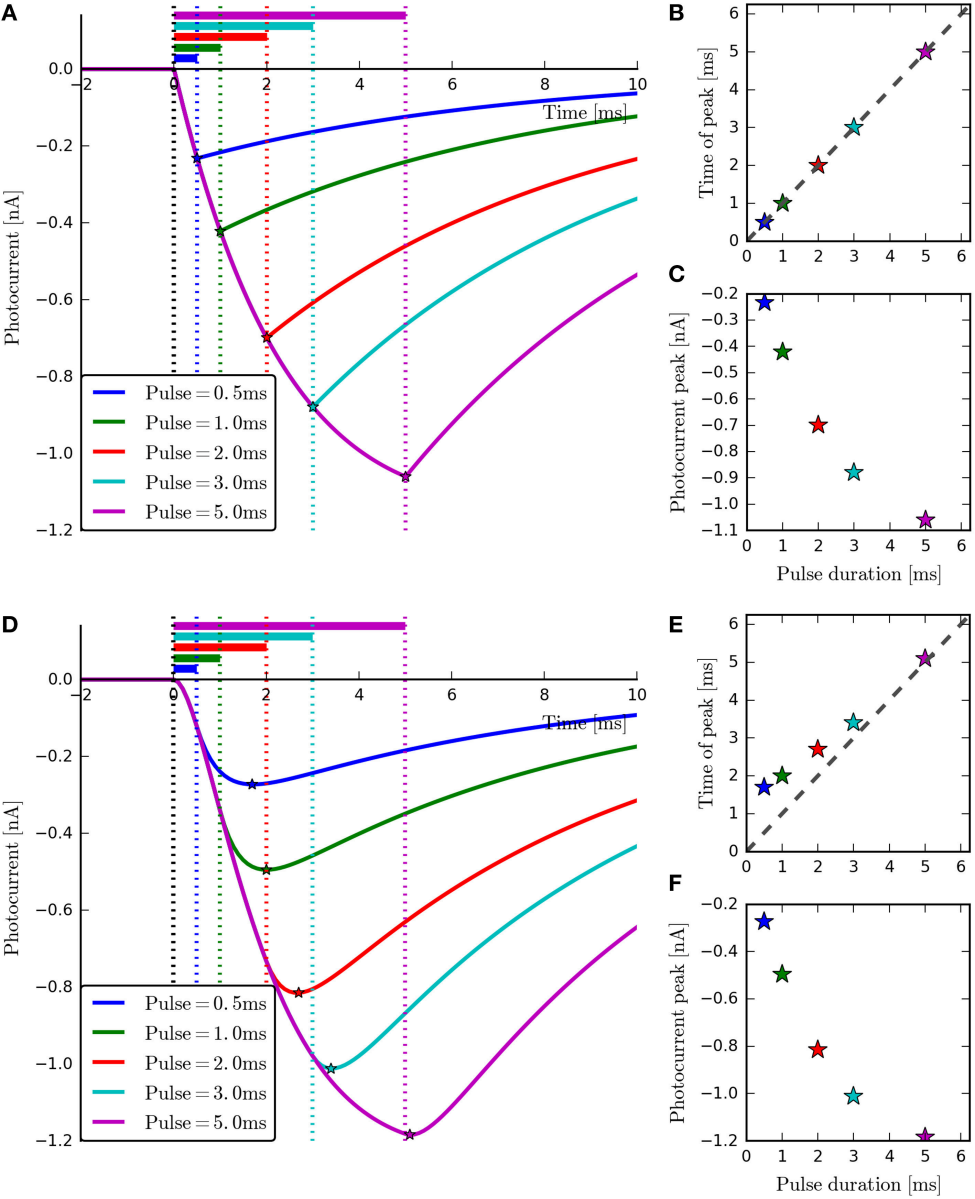
**The four-state and six-state models simulated with ChR2-derived parameters for short-duration pulses**. With the four-state photocurrents **(A)**, the peak occurs at the end of the on-phase. This can be seen most clearly in the plot of Pulse duration vs. Time of peak **(B)** where all points lie on the diagonal. For the six-state photocurrents however, the peaks lag behind the end of the illumination periods slightly **(D)**, due to the transitions to and from the model's extra intermediate states. The inactivation phases **(A,D)** and magnitude of the peaks **(C,F)** can be seen to be very similar for both models. For the six-state model, the peak-lag effect can also be observed to diminish as the pulse duration increases **(E)**, demonstrating the practical equivalence of the two models for long stimulation periods.

All light pulses should be “rectangular” (step functions) in that they have a sharp onset and offset. Examples of each protocol are included in PyRhO with illustrations provided in Figures 3–**7**. The duration of the on- and off-phases should also be kept approximately equal since the optimizer will effectively weight the contributions of each according to the relative numbers of data points. Additional parameter dependencies will be added in the future which may require additional data sets for a full characterization of the models.

#### 2.5.2. Minimal data requirements

In general, the most important data are those described for characterizing the flux dependence, which may be considered to be the “minimal set.” If this set consists of only a single photocurrent, the fitting algorithms will fix the parameters which model the flux dependence (ϕ_*m*_, *p* and *q*) to the initial values supplied (along with fixing those describing other uncharacterized variables) and tune the remaining parameters to return a model fit for that specific flux. This is not recommended however, as the model is under-constrained by the data (typically resulting in a poorer fit than when using a whole set of photocurrents) and is unlikely to generalize well to new experimental conditions. For best results, the flux dependence photocurrents should be measured at light intensities spanning several orders of magnitude as described above.

If variations in other parameters or short pulses are of interest then the additional data should (ideally) be collected as described. However, if obtaining the data for a full characterization of the model is not possible, the pre-set default values should be adequate for most practical purposes.

#### 2.5.3. Data format

Each voltage-clamp recorded photocurrent should be loaded into a PhotoCurrent object as follows:

pc = PhotoCurrent(I=i0, t=t, pulses=[[t_on,
                  t_off]], phi=2e15, V=-70)


Here, I is the array of photocurrent values in nanoamperes, t is the corresponding array of recording times (or a scalar representing the time-step) in milliseconds, pulses is a nested list of *n* lists (or an *n* × 2 array), where *n* corresponds to the number of pulses and each inner list contains the on-time and off-time for each pulse in milliseconds, phi represents the stimulating flux value in photons · mm^−2^ · s^−1^ and V is the clamp voltage in millivolts (or “None” if the voltage was not clamped).

The PhotoCurrent class contains methods which automatically check the data and extract the key features from it, which may then be accessed as properties of the object with the . operator. Any properties which are data-derived are suffixed with “_,” for example, the peak and steady-state current are accessed with pc.peak_ and pc.ss_, respectively. These photocurrents may easily be plotted, along with their main features and the light stimulus using the plot() method.

The PhotoCurrent objects are then combined into ProtocolData objects. These sets of photocurrents also provide several convenient methods for plotting and extracting parameters from the data set as a whole.

  stepPD = ProtocolData(protocol="step",
     nRuns=1, phis=[1e14,1e15,1e16,1e17,1e18],
                                    Vs=[-70])
  
  for iPhi, phi in enumerate(phis):
      for iV, V in enumerate(Vs):
          pc = PhotoCurrent(Is[iPhi][iV], t,
                            pulses, phi, V) 
          stepPD.addTrial(pc)


Finally, the data sets are combined into a dictionary using the protocol names as keys:


  ChR2DataSet = {"step" : stepPD,
              "recovery" : recovPD,
              "rectifier" : rectiPD,
              "shortPulse" : shortPD}


This dictionary contains all the data necessary to parameterize all three models, however, if only the three and four-state models are of interest then the "shortPulse" protocol may be omitted.

#### 2.5.4. Fitting procedure and algorithms

Once the data have been loaded into the appropriate structures, the fitting algorithms may be called with the fitModels() function.


fp = fitModels(ChR2dataSet,  nStates=6,
               params=initialParams)


This procedure returns a Parameters object (from the lmfit module) with the calculated values and plots the resultant model fits over the experimental photocurrents. The entire set of ChR2 data are shown fitted to the six-state model for each flux value spanning two orders of magnitude (ϕ = [2.21 × 10^15^, 2.65 × 10^17^] photons · mm^−2^ · s^−1^) with the same set of parameters in Figure 5. The lowest and highest intensity photocurrents are also shown in Figure 6 with the model fits for both the three- and four-state models for direct comparison. The six-state model fits are not replotted here as they only exhibit a significant difference to the four-state fits for short pulses, as illustrated in Figure 7.

**Figure 8:**
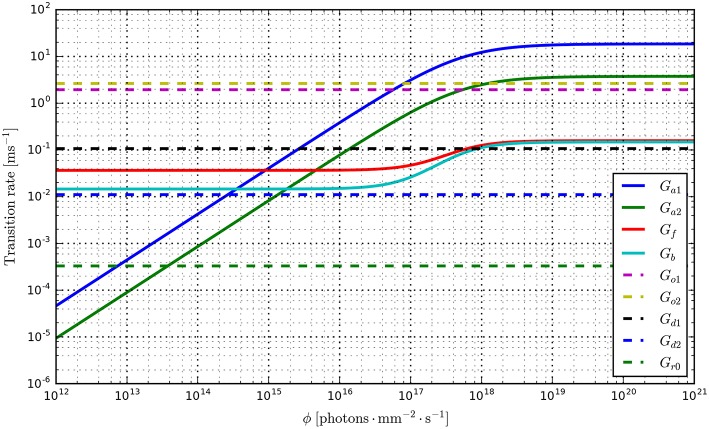
**Transition rate plots for the six-state model fit to ChR2 data (on log-log axes) where light-dependent transitions are shown with solid lines and light-independent transitions are shown with dashed lines**.

**Figure 9:**
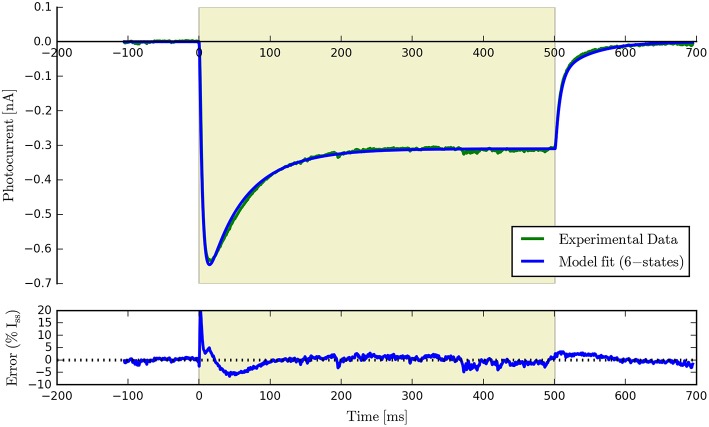
**An example six-state model fit to ChR2 data (at ϕ = 2.21 × 10^15^ photons · mm^−2^ · s^−1^) with the accompanying residual error expressed as a percentage of the steady-state current**.

**Figure 10:**
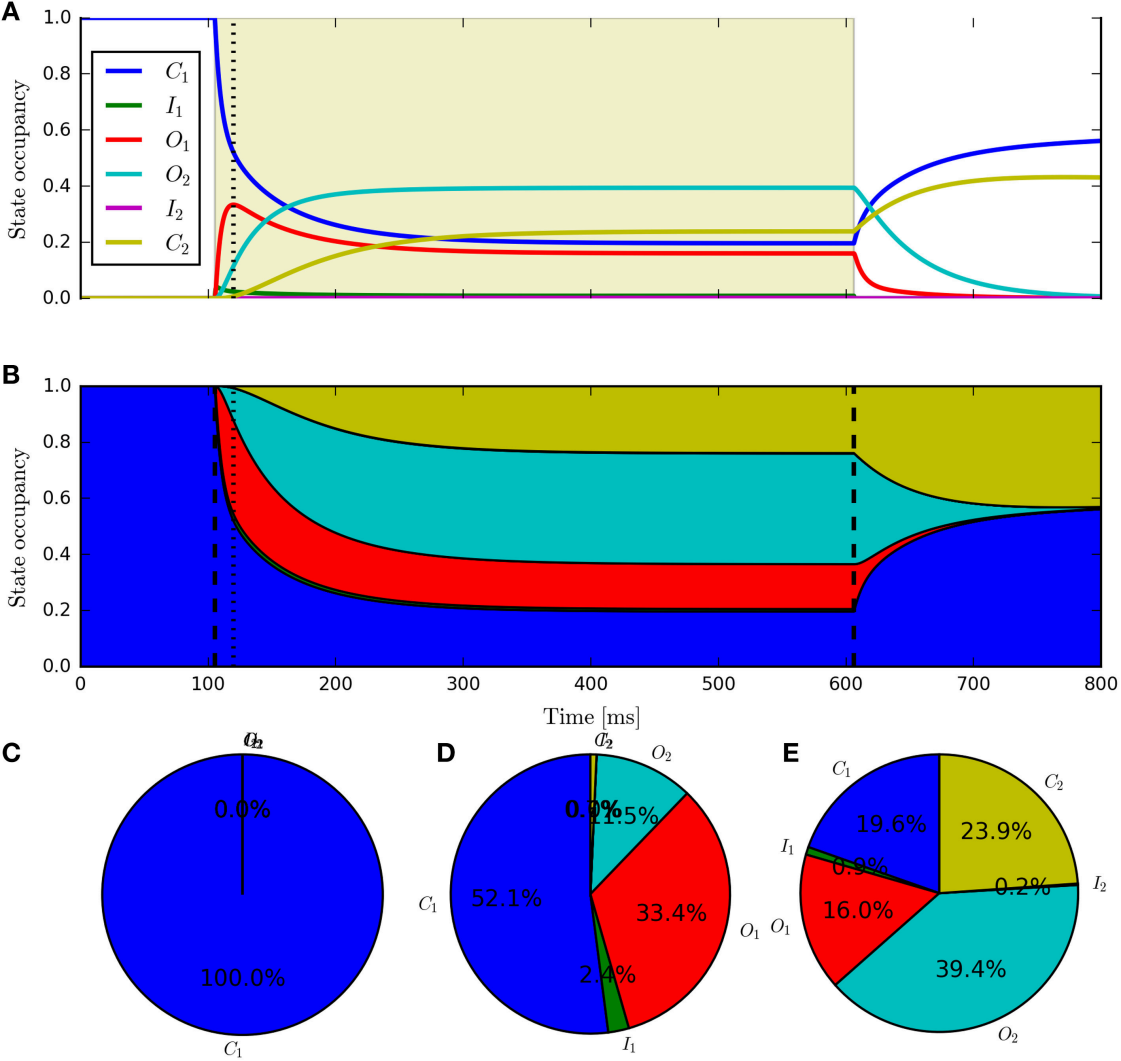
**The six-state model's internal states**. This figure corresponds to the example fitting plot (Figure 9) and shows the evolution of the internal states through time **(A,B)** shows alternative representations of the same data) along with the occupancy proportions at the initial conditions **(C)**, peak **(D)** and steady-state **(E)**.

Having fit a model, it may be easily characterized by plotting how the light-dependent transition rates vary as a function of flux (based on the Hill equation) along with light-independent transition rates as shown in Figure 8. An individual fit is shown in more detail with the residual error for ϕ = 2.21 × 10^15^ photons · mm^−2^ · s^−1^ in Figure 9. To provide insight into the model's kinetics, PyRhO also offers state variable plots. The evolution of the six-state model corresponding to the fit in Figure 9 is given in Figure 10.

In general terms, the fitting algorithm first finds the model-independent variables such as the dark recovery rate and voltage dependence factors, proceeding through “off-curve” parameters by fitting a double exponential decay function, optionally fitting opsin activation rates for the six-state model and finally optimizing across a set of “on-curves” to find any remaining parameters. Due to the inherent variability and imprecision in experimental measurements there is an optional second optimization phase over the entire set of photocurrents simultaneously. The values found for the dark parameters {*G*_*d*(1, 2)_, [*G*_*f*0_, *G*_*b*0_]} (and opsin activation rates *G*_*o*(1, 2)_ if relevant) are used as the initial values, lower and upper bounds are calculated as 50 and 200% of these values, respectively (set by a hyperparameter) and the model is then re-optimized to achieve an overall better fit. The main sub-routines of the algorithm are given in Algorithm 1 with more detail for each process given in the Appendix.

**Algorithm 1:** **Fitting algorithm**.

1: **function** fitModel({*DataSet*}, {*initParams*})
2: **if** "*rectifier*" in {*DataSet*} **then**
3: (*E*, *v*_0_, *v*_1_) ← fitVoltageRectifier(**V_c_**, **I_ss_**)
4: *g*_0_ ← fitConductance(*v*, *E*, max_ϕ_(**I_p_**))
5: **if**"*recovery*" in {*DataSet*} **then**
6: *G*_*r*0_ ← fitPeakRecovery(**t_p_**, **I_p_**)
7: (*G*_*d*(1,2)_, [*G*_*f*0_, *G*_*b*0_]) ← fitOffCurves({*I*_ϕ_[*t*_*off*_:]})
8: (ϕ_*m*_, *k*_(1,2)_, *p*, [*G*_*r*1_, *G*_*f*0_, *k_f_*, *q*, *G*_*b*0_, *k_b_*, γ, *G*_*o*(1,2)_])
← fitOnCurves({*I*_ϕ_[*t_on_* : *t_off_*]})
9: **if** postFitOptimization is True **then**
10: ({*All parameters*}) ← fitCurves({*I*_ϕ_})

When fitting the three-state model, a double exponential is fit (with two corresponding decay rates *G*_*d*1_ and *G*_*d*2_) which are then weighted by their coefficients (*I*_slow_ and *I*_fast_) and combined to form a single exponential. The mean of these values is then calculated across a set of *N* photocurrents (Equation 7) and this value is then used in subsequent parts of the fitting algorithm.

(7) $$\begin{array}{r} G_{d}=\frac{1}{N}\overset{N}{\underset{n=1}{\sum }}\frac{I_{{slow}_{n}}\times G_{d1_{n}}+I_{{fast}_{n}}\times G_{d2_{n}}}{I_{{slow}_{n}}+I_{{fast}_{n}}}\;. \end{array}$$

#### 2.5.5. Verification

In order to test the algorithms, synthetic data was generated using the parameter values derived from fitting the six-state model to the ChR2 experimental data. The fitting procedure was then applied to these synthetic photocurrents to compare the newly derived parameters with the known values used to generate the synthetic data. The results of these two fitting processes using the “powell” optimization algorithm are shown in Table 3 (along with the values used as initial estimates).

**Table 3:** **Comparison of parameters found in fitting to those used to generate the fitting data**.

**Parameter**	**Initial**	**Experimental**	**Computed**	**Difference**
*g*_0_ (*pS*)	2.5e4	2.76e+04	2.77e+04	+0.0464%
γ (1)	0.05	8.33e-16	0.00369	Δ: +0.00369
ϕ_*m*_ (*ph*. · *mm*^−2^ · *s*^−1^)	3.5e17	5.07e+17	5.02e+17	−0.983%
*k*_1_ (*ms*^−1^)	10	18.5	18.2	−1.3%
*k*_2_ (*ms*^−1^)	3	3.75	4.07	+8.68%
*p* (1)	1	0.982	0.981	−0.0921%
*G*_*f*0_ (*ms*^−1^)	0.04	0.0365	0.0365	+0.149%
*k*_*f*_ (*ms*^−1^)	0.1	0.121	0.121	−0.601%
*G*_*b*0_ (*ms*^−1^)	0.02	0.0146	0.0143	−1.99%
*k*_*b*_ (*ms*^−1^)	0.15	0.133	0.131	−1.31%
*q* (1)	1	1.45	1.45	−0.196%
*G*_*o*1_ (*ms*^−1^)	2	1.93	1.93	+0.0256%
*G*_*o*2_ ([*ms*^−1^)	2	2.65	3.38	+27.7%
*G*_*d*1_ (*ms*^−1^)	0.1	0.108	0.108	+0.453%
*G*_*d*2_ (*ms*^−1^)	0.01	0.0111	0.0115	+3.96%
*G*_*r*0_ (*ms*^−1^)	0.00033	0.00033	0.00033	−0.0585%
*E* (*mV*)	0	0	3.9e-08	Δ: +3.9e-08
*v*_0_ (*mV*)	43	43	43	−2.16e-08%
*v*_1_ (*mV*)	17.1	17.1	17.1	+0.00889%

We first note that many of the computed parameters are very close to the true (original) values (all but two are within ±5% of the original values), especially in the context of the degree of experimental noise and measurement error which would typically accompany recordings of real neuronal data. One notable exception is *G*_*o*2_ which is hard to fit in a single-pulse protocol since all opsin models are assumed to be in their fully dark-adapted (ground) state i.e., *C*_1_ = 1.

**Figure 11:**
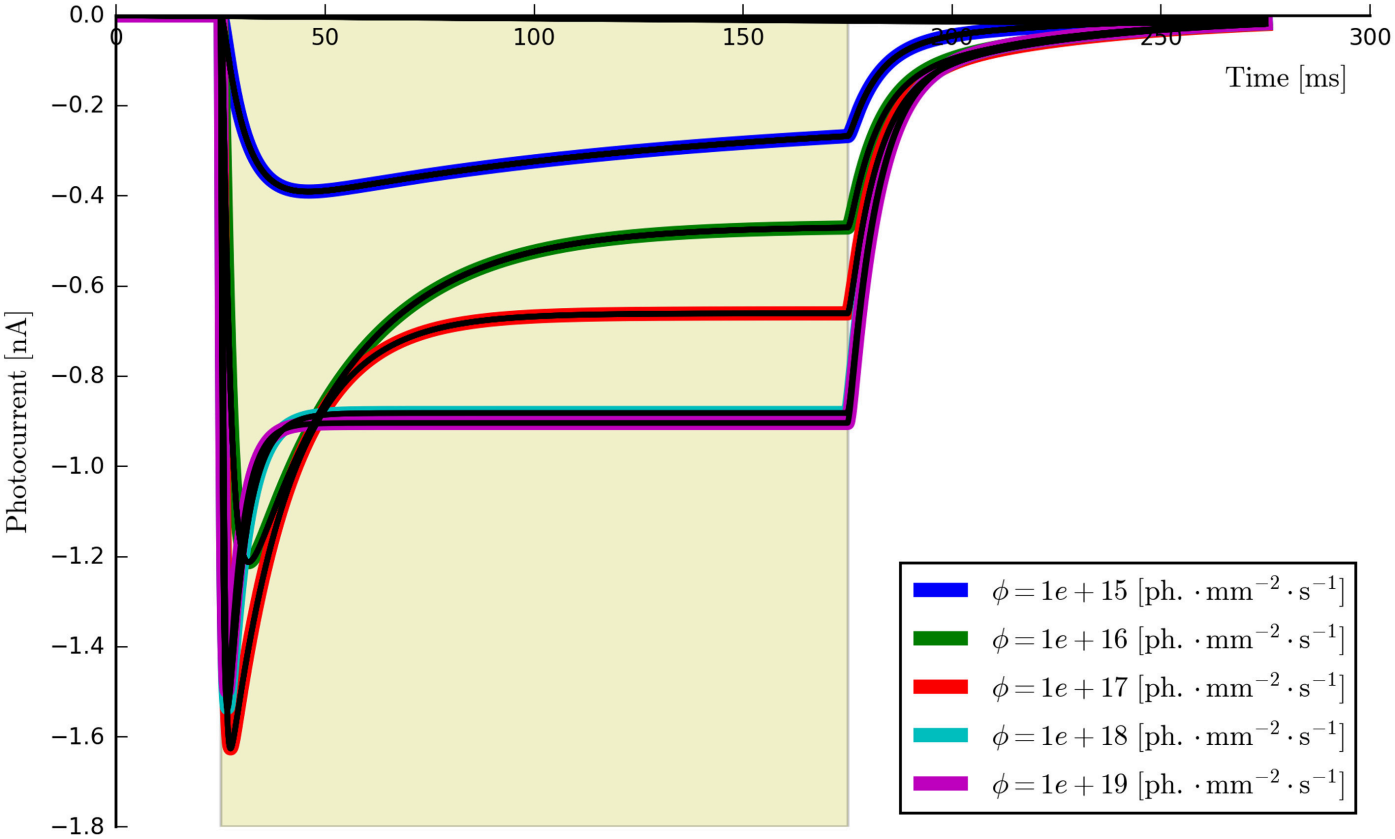
**The six-state model (re-)fitted to synthetic data generated from parameters derived from six ChR2 photocurrents**. It can be seen that the model fits (black lines) almost perfectly fit each synthetic photocurrent using a unified parameter set.

While there are other differences between some of the original and computed parameters, these may potentially be accounted for by numerical precision issues and the high-dimensional parameter space of the six-state model being under-constrained by the data. For example, a decrease in one parameter may be compensated for by an increase in another such that only latent variables are affected and the fit in the observable current is still good. Inspecting the model fit plots appears to confirm this, as the residual error is very low across the whole set of generated verification photocurrents—at most ±0.5% of the steady-state current and usually considerably less. The entire set of photocurrents fitted to the synthetic data are plotted in Figure 11 and show a very close correspondence to the target (synthetic) photocurrents across the whole set of stimulus intensities.

### 2.6. Computational simulation

#### 2.6.1. Simulations procedure

To programmatically simulate the opsin, a model, protocol and simulator object must first be created (see Figure 1). The model and protocol are then loaded into the simulator which configures the simulation environment for that particular choice of opsin and protocol (e.g., by setting the numerical time-step according to the shortest stimulation period). The simulator object can then be run and plotted with the appropriate methods as shown in the following example, where a six-state model is used with the ChR2 parameters and run through the rectifier protocol (described below). In this example the protocol is run on the NEURON simulator, (rather than the default Python simulator) and the default ChR2 parameters are loaded from the module's modelFits dictionary, which contains some pre-fit model parameter sets for several common opsins.


  from pyrho import *
  
  nStates = "6"       # 3, 4 or 6
  ChR2params = modelFits[nStates]["ChR2"]
  RhO = models[nStates](ChR2params)
  Prot = protocols["rectifier"]()
  Sim = simulators["NEURON"](Prot, RhO)
  
  Sim.run()
  Sim.plot()
 


Alternatively, when using the PyRhO GUI, the model, protocol and simulator are simply selected from drop-down lists, (optionally parameters may be changed), then simulated and plotted by clicking the “Run” button.

Each type of object will be initialized with default parameters (in the form of Parameters objects) unless passed a different set upon initialization e.g., RhO = models[nStates](params6s). Alternatively, parameters may be set after creation using methods such as .setParams() or .updateParams() for partial sets.

#### 2.6.2. Protocols

PyRhO comes with several preconfigured and customizable simulation protocols for exploring the dynamics of the models. These include typical system analysis stimuli such as delta functions, step functions and sinusoids, as well as chirps and specialized protocols designed to probe particular features of the opsins including voltage-dependence (rectifier), opsin activation (shortPulse) and dark recovery (recovery).

#### 2.6.3. Simulators

PyRhO's simulation layer serves to perform house-keeping tasks necessary to prepare different simulation environments to use a particular opsin model in a “system” of interest and apply a particular protocol to it. Currently, three simulators are available in PyRhO: Brian2 for neural networks, NEURON for detailed morphological neurons and (pure) Python for basic opsin channel dynamics. This selection of simulators provides PyRhO with a way to seamlessly span multiple scales of modeling with the same parameterized opsins; from individual channels to whole brain regions.

When using simulators other than “Python,” additional parameters may be specified. For example, the NEURON simulator has additional parameters such as “v_init” (the cell membrane potential initialization value), “CVode” (a boolean value for activating variable time-step solvers), and “cell” (a hoc file specifying the neuron's morphology). This allows existing simulations created in NEURON to be conveniently transfected (augmented with opsins) and run within the PyRhO framework. While models may be fit to data and then seamlessly inserted into one of these simulators within the same environment, if desired the NMODL files and Brian equations may be accessed and exported as a starting point for creating stand-alone simulations.

**Figure 12:**
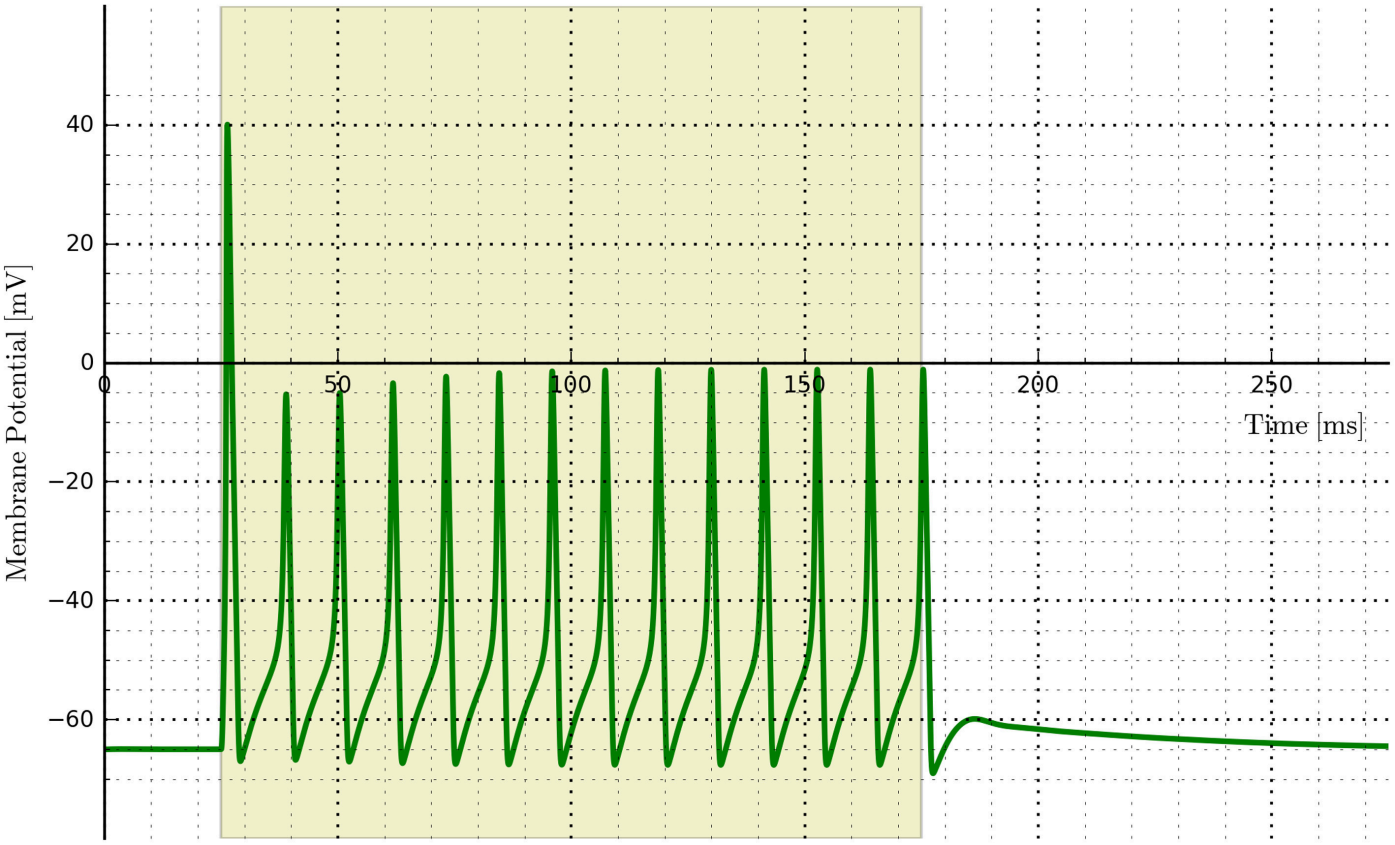
**Cell membrane potential for a simple Hodgkin–Huxley neuron transfected with a six-state opsin model of ChR2 and simulated in NEURON**.

An example of implementing opsins within the NEURON environment is shown in Figure 12 where ChR2 expressing cells are illuminated with a 150 ms light pulse. The six-state equations were used to model the ChR2 additions and implemented via the NMODL file RhO6.mod, adapted from the description in Grossman et al. (2013).

**Figure 13:**
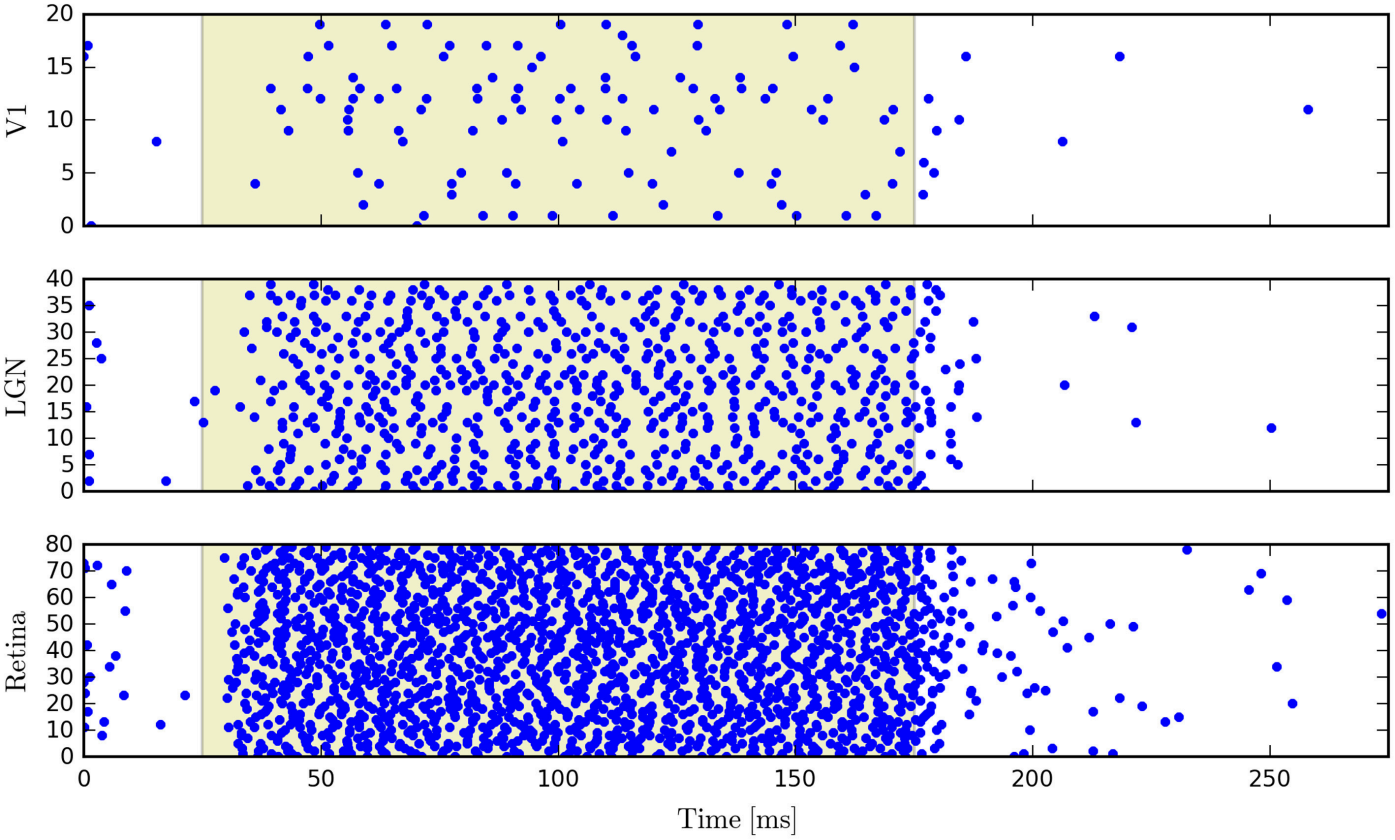
**Raster plots for a three-layer network of leaky integrate-and-fire neurons simulated with Brian2 where the first layer has been transfected with a six-state model of ChR2**.

PyRhO also incorporates the Brian2 spiking neural network simulator. The opsin is represented with a set of ODEs which use the parameters specified in the RhodopsinModel object. As an example we show simulation results for a neural network which consists of 140 leaky integrate-and-fire neurons, separated into three feed-forward layers. The first group has neurons which express ChR2 and that layer has a set of random connections with the next layer, with 20% connectivity and 1*ms* conduction delays (with the same specifications for connectivity between the second and third layers). Figure 13 shows raster plots of the spiking neurons in all three layers.

### 2.7. Graphical user interface

To make PyRhO usable without any programming background, a graphical user interface (GUI) has been written which runs in Jupyter (formerly IPython; Pérez and Granger, 2007). This runs in a browser-based notebook meaning that it could be easily configured as a server and made accessible to an entire laboratory or classroom without requiring local installations on each machine. Since both figures and text results appear embedded in the notebook after the code used to produce them, this makes the interface self-documenting and a particularly useful means of sharing models (Topalidou et al., 2015).

**Figure 14:**
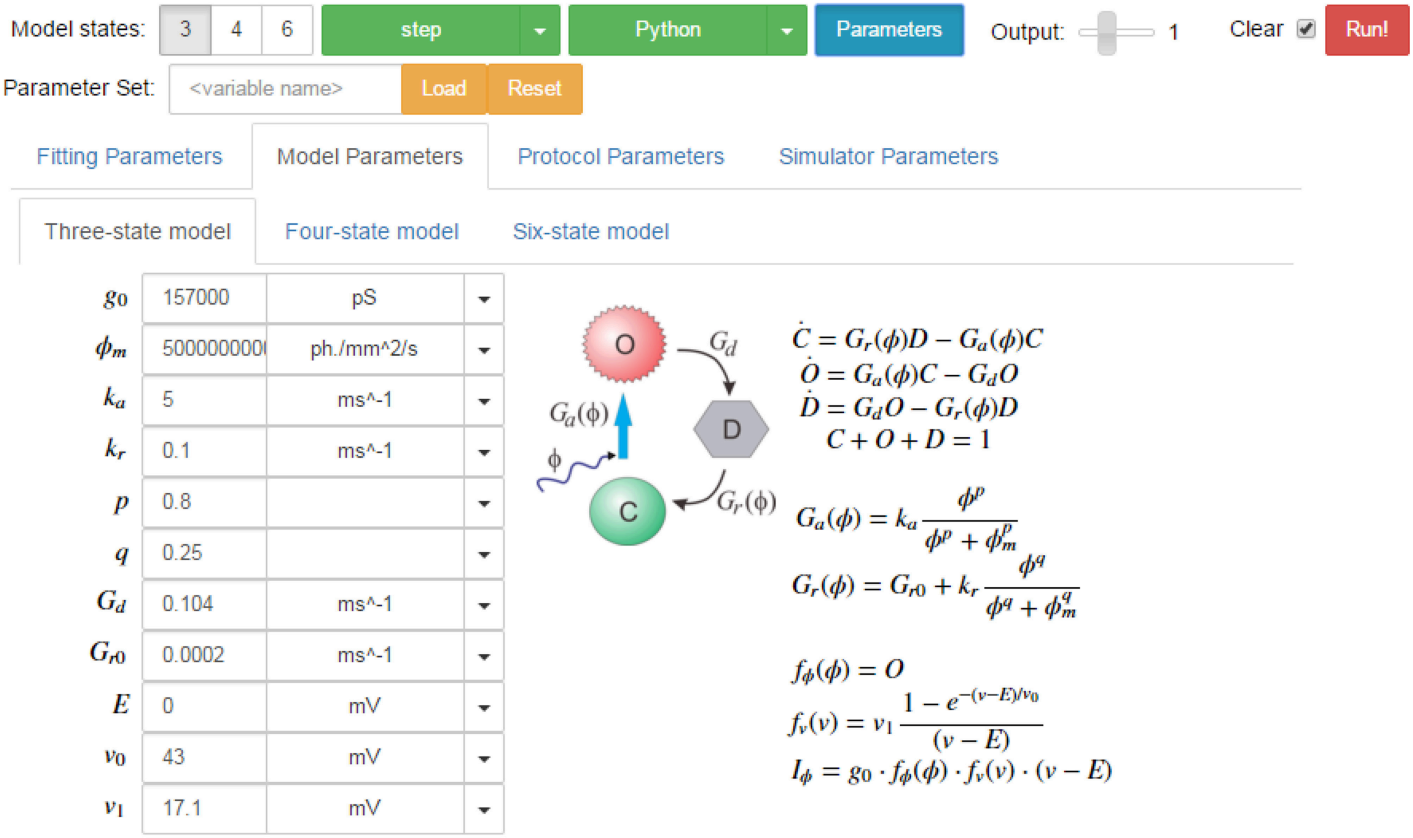
**Screen shot of the PyRhO GUI (in expanded view) showing the run bar at the top for running simulations and the models tab below where parameters may be adjusted**.

A screenshot of the GUI is given in Figure 14 showing the simulation tab for the three-state model. In addition to the parameter fields, the model states diagram is also embedded in the GUI with the equations which describe the opsin's behavior rendered in LaTeX. Similarly, the parameters for each protocol and each simulator are shown on other tabs for easy modification of their values. On the fitting tab, there are also sub-tabs for each of the models, with their respective sets of parameters including tick-boxes to fix parameters and fields for numerical bounds and algebraic constraints to be passed to the fitting algorithms.

## 3. Discussion

PyRhO has been written to be an integrated suite of intuitive, flexible, open-source and multi-scale computational tools for analysing and simulating opsins. In keeping with the open-source community's ethos, it builds upon existing libraries for numerical (NumPy), scientific (SciPy, lmfit) and plotting routines (Matplotlib). It also incorporates several freely available simulators, namely NEURON and Brian2, with work to incorporate PyNEST underway.

### 3.1. Classes of opsin

While the models were developed with rhodopsins and the fitting and simulation demonstrations are illustrated using ChR2 data, in principle the tools should work just as well with other classes of opsin. Essentially the opsin models represent non-linear dynamical systems (second order for the three-state model and *n* − 1^*th*^ order for the *n*-state model in general). As such they are capable of capturing the three main classes of dynamics in response to a step input: under-damped, over-damped and oscillatory (ringing) currents. While PyRhO can fit and simulate all three cases, interestingly, only the first two types of response have been observed so far (for low and high intensity stimulation, respectively).

For inhibitory opsins observed so far, while the sign of the current changes, the dynamics remain qualitatively the same, meaning that the fitting and simulation routines should be just as effective. However, for fitting, it may be necessary to adjust the starting values for some parameters to help the algorithms achieve good results. The main changes we anticipate would be in parameters that are extraneous to the the core dynamics (that is, those not described by the differential equations) such as the reversal potential, (*E*) which may be measured through standard electro-physiological techniques and possibly the parameters tuning the voltage rectification curve (*v*_0_ and *v*_1_).

### 3.2. Extensibility

Given the way that PyRhO has been constructed around several layers of abstraction, extending the capabilities with more models, simulators or protocols is relatively straightforward. Nested classes (and sub-classes) act as templates guiding development while inheritance of methods and attributes facilitates code reuse in line with open-source software development best practices. As more data is collected, the library of parameterized models may be contributed to, making more characterized opsin variants available for other optogeneticists to simulate.

In broader terms, PyRhO has potential as a framework to be generalized to incorporate other types of stimulation, including for example electrical and magnetic. The simulator and protocol layers could potentially remain largely unaltered allowing it to relatively easily grow into a neural stimulation platform with neuro-engineering applications beyond the scope of just optogenetics.

### 3.3. Limitations

The fitting algorithms rely upon optimization methods to extract several parameters and hence are subject to the standard issues associated with such procedures, including sensitivity to initial conditions and settling in local minima. To ameliorate these issues, the lmfit module is used to provide several useful facilities including imposing bounds on the parameters and algebraic constraints. Individual parameters may also be fixed and the fitting procedures rerun to optimize over the remaining free parameters, possibly with a new set of initial conditions. Measuring and then fixing physiological parameters in this way, such as the reversal potential *E*, will help the optimization algorithms and considerably improve the resultant model fit.

Additionally there are limitations due to the more specific nature of the models used. For example, the routine for estimating *g*_0_, the biological scaling parameter for the cell's conductance, is systematically under-estimated. This should be the maximum conductance of the cell (voltage clamped to −70 mV) assuming full occupancy of the (primary) open-state. However, in simulations this condition is never achieved in the models, with maximum occupancy dependent on the model parameters, typically found to be around 0.8 for wild-type ChR2 (Nikolic et al., 2009). We therefore calculate a conservative correction factor to yield a better (albeit imperfect) estimate and make the user aware of the issue so that they may override and fix the values returned from the fitting function as necessary.

We note however that these limitations are not major, especially in the context of experimental and measurement inaccuracies and so do not significantly detract from the usefulness of PyRhO's fitting algorithms.

### 3.4. Future work

One natural development for PyRhO would be to extend the models (and fitting algorithms) to include additional parameter dependencies defined in the introduction, Equation (3), such as spectral absorption *f*_λ_(λ), temperature *f*_T_(*T*|*Q*_10_), and pH *f*_pH_(*pH*_*int*_, *pH*_*ext*_) factors for the channel conductance, as well as the effects of temperature and pH on the photocycle kinetics. This would allow the simulations to capture more of the variation and enhance the tools' ability to engineer the response to a target outcome or compensate for adverse experimental conditions.

The unconstrained nature of the parameter *G*_*o*2_ also suggests that there may be scope for additional models. For example, a simpler five-state model (similar to the six-state model but without *I*_2_ and its associated transitions) may be able to capture the experimental data as well as the six-state model with a less arduous fitting process and more stable resulting parameters (less sensitive to initial choices). In the first release however, we have used the six-state model for the sake of greater generality.

Another route for future development would be to incorporate other simulators for example PyNEST (Eppler et al., 2008), allowing greater flexibility for users to choose the simulator they are most comfortable with or which offers alternative features and performance benefits. Ultimately this process could be continued to interface with PyNN (Davison et al., 2009) and other simulator-agnostic model description languages such as NeuroML (Gleeson et al., 2010) and NineML (Gorchetchnikov et al., 2011). Furthermore, PyRhO could be combined with the software for optical pattern generation and data acquisition NeuroPG (Avants et al., 2015), to create a complete neural engineering tool for optogenetics.

While there is always scope to add additional protocols, a particularly interesting approach may be to allow networks of neurons to be transfected with several types of opsin and stimulated with multiple wavelengths of light (Han and Boyden, 2007). This could also be supplemented with a more detailed model of the optics of light-tissue interactions as previously implemented for an individual cell in NEURON (Foutz et al., 2012) or interfaced with OptogenSIM for whole brain simulations (Liu et al., 2015). These would be particularly useful additions to PyRhO's neuro-engineering capabilities, allowing stimuli to be more accurately sculpted. In the meantime, users of PyRhO should be mindful of the attenuating effects of light scattering and absorption (or equivalently a spectral shift) from passing through other tissue (particularly *in vivo*) which are not currently explicitly accounted for. These effects may result in a lower effective flux intensity than specified which may shift the “true” value of ϕ_*m*_ recovered from the fitting procedures.

In summary, we have presented and verified a new integrated suite of open-source computational tools for optogenetics. PyRhO has been demonstrated to characterize opsins from experimental photocurrents, fitting kinetic model parameters to yield a functional understanding, helping to guide opsin choice and development. These models have been demonstrated in simulations across multiple scales, from channels to networks, by harnessing popular simulators such as NEURON and Brian2. PyRhO is also provided with a Jupyter browser-based GUI to facilitate its use and aid in model sharing. We have outlined some of its chief strengths along with its limitations and plans for future improvements. By releasing these tools as open-source, we hope that other computational neuroscientists will contribute features and expertise, accelerating progress in the rapidly growing field of optogenetics.

## Author contributions

BE designed and wrote the software module and GUI. KN and BE developed the opsin models. BE and KN developed the model fitting algorithms. BE primarily wrote the article, ran the tests, analyzed the data and plotted the figures with participation from KN. BE, KN, SJ, and SS contributed to the project concepts, manuscript comments and discussion of results.

## Funding

This work was supported by the UK Biotechnology and Biological Sciences Research Council (BBSRC) grant BB/L018268/1 and the UK Engineering and Physical Sciences Research Council (EPSRC) grant EP/N002474/1.

### Conflict of interest statement

The authors declare that the research was conducted in the absence of any commercial or financial relationships that could be construed as a potential conflict of interest.

## Appendix

### Three-state analytic solution

The analytic solution for arbitrary initial conditions (*C*_0_, *O*_0_, *D*_0_) is as follows:

(A1) $$\begin{array}{l} C(t)=\;\frac{G_{d}G_{r}}{\lambda_{1}\lambda_{2}}+\frac{\left(\lambda_{1}-G_{d}-G_{r}\right)}{G_{d}\lambda_{1}\xi }\;\cdot \;\zeta_{1}\;\cdot \;e^{-\lambda_{1}\cdot t}\\ \;-\;\frac{\left(\lambda_{2}-G_{d}-G_{r}\right)}{G_{d}\lambda_{2}\xi }\;\cdot \;\zeta_{2}\;\cdot \;e^{-\lambda_{2}\cdot t} \end{array}$$

(A2) $$\begin{array}{l} O(t)=\;\frac{G_{a}G_{r}}{\lambda_{1}\lambda_{2}}-\frac{\left(\lambda_{1}-G_{r}\right)}{G_{d}\lambda_{1}\xi }\;\cdot \;\zeta_{1}\;\cdot \;e^{-\lambda_{1}\cdot t}\\ \;-\;\frac{\left(\lambda_{2}-G_{r}\right)}{G_{d}\lambda_{2}\xi }\;\cdot \;\zeta_{2}\;\cdot \;e^{-\lambda_{2}\cdot t} \end{array}$$

(A3) $$\begin{array}{l} D(t)=\;\frac{G_{a}G_{d}}{\lambda_{1}\lambda_{2}}+\frac{1}{\lambda_{1}\xi }\;\cdot \;\zeta_{1}\;\cdot \;e^{-\lambda_{1}\cdot t}\\ \;-\;\frac{1}{\lambda_{2}\xi }\;\cdot \;\zeta_{2}\;\cdot \;e^{-\lambda_{2}\cdot t} \end{array}$$

with the following definitions:

$$\xi =\sqrt{G_{a}^{2}+G_{d}^{2}+G_{r}^{2}-2\cdot \left(G_{a}G_{d}+G_{a}G_{r}+G_{d}G_{r}\right)}$$

$$\lambda_{1,2}=\frac{1}{2}\cdot \left(\left(G_{a}+G_{d}+G_{r}\right)\pm \xi \right)$$

$$\begin{array}{rcl} \zeta_{1,2} & = & C_{0}\cdot \left[G_{d}\cdot G_{a}\right]+O_{0}\cdot \left[G_{d}\cdot \left(G_{a}-\lambda_{1,2}\right)\right] \end{array}$$

$$\begin{array}{r} +\;D_{0}\cdot \left[G_{r}\cdot \left(\lambda_{1,2}-G_{a}-G_{d}\right)\right] \end{array}$$

Note also that λ_1_ · λ_2_ = *G*_*a*_*G*_*d*_ + *G*_*a*_*G*_*r*_ + *G*_*d*_*G*_*r*_ i.e., the sum of products.

### Model-independent fitting procedures

Prior to fitting the specific parameters of a chosen model, several fitting processes are performed to find parameters common to all models:

1. If a set of voltage clamp data are provided over a range of potentials, the steady-state currents are used to fit the rectification function, *f*_v_(*v*), (Equation 5) yielding the parameters *E*, *v*_0_, and *v*_1_:
  - The *I*_*ss*_ values are first fit to the function *f*_v_(**V_clamp_**) · (**V_clamp_** − *E*) to find an initial estimate for *v*_0_ and a value for *E* where the polarity of the steady-state currents changes (along with a dummy parameter comprising *g*_0_ · *f*_ϕ_ · *v*_1_, which is discarded).
  - The reversal potential, *E*, is fixed and the steady-state currents are converted to conductances: **g** = **I_ss_ ∕ (V_clamp_** − *E*). These conductances are then normalized by dividing by the conductance calculated at *V*_*clamp*_ = −70 mV to produce an array of conductance factors.
  - The conductance factors are fit to the function *f*_v_(*V*_*clamp*_) with the constraint $v_{1}=\left(\mathrm{70}+E\right)∕\left(e^{\frac{70+E}{v_{0}}}-1\right)$ to calculate and fix the parameters *E*, *v*_0_ and *v*_1_.
2. All photocurrents in the dataset recorded at −70 *mV* are scanned to find the highest peak. This value is used to calculate an initial estimate for *g*_0_ (according to the formula *g*_0_ = *I*_peak, max_ ∕ (−70 − *E*)) to aid the optimization routines.
3. If the recovery protocol is included in the dataset (pairs of pulses with a range of inter-pulse-intervals) then the peak currents of the second pulses are extracted along with the times and the initial peak and steady-state current. These values are then aligned to the end of the first pulse (where the current is *I*_*ss*0_) and fit to the exponential recovery function: $I_{\text{peak}}\left(t\right)=I_{peak0}-a\cdot e^{G_{r0}\cdot t}$. This yields the dark recovery rate constant, *G*_*r*0_.

These parameters are then fixed (except for the estimate of *g*_0_) and passed to the model-specific fitting routines.

### Model-dependent fitting procedures

Each model is fit with broadly the same three-stage fitting procedure consisting of Off-curve parameters, then On-curve parameters and finally re-optimizing:

1a. Fit bi-exponential functions to the Off-curves to find parameter values for the light-insensitive decay rates: {*G*_*d*1_, *G*_*d*2_, *G*_*f*0_, *G*_*b*0_} for the four- and six-state models [detailed in Section Four- and Six-state Model Off-curve Fitting (Step 1a.)], or *G*_*d*_ for the three-state model (described in Equation 7),
1b. [Six-state only] Fix the values for the parameters from Step 1a then calculate activation rates *G*_*o*1_ and *G*_*o*2_ [detailed in Section Six-State Opsin Activation Rate Fitting (Step 1b.)],
2. Fix the values for the parameters found in Step 1 and fit the On-curves to find the remaining parameters governing the light-dependent transitions,
3. Using the values found in Steps 1 and 2 as initial values, relax all parameter values to freely vary within a range (by default between 50 and 200% of the initial values) and refit the model across the set of whole photocurrent curves (on- and off-phases).

#### Four- and six-state model off-curve fitting (step 1a.)

The fitting equations for the Light-Off response curves can be calculated directly from the set of ODEs given in Table 2, for light flux zero, as shown in Nikolic et al. (2009):

(A4) $$\begin{array}{rcl} O_{1\text{off}} & = & A_{11}\exp\left(-\Lambda_{1}t\right)+A_{12}\exp\left(-\Lambda_{2}t\right) \end{array}$$

(A5) $$\begin{array}{rcl} O_{2\text{off}} & = & A_{21}\exp\left(-\Lambda_{1}t\right)+A_{22}\exp\left(-\Lambda_{2}t\right) \end{array}$$

where

$$\Lambda_{1,2}=b\mp c\;,$$

*b* = (*G*_d1_ + *G*_d2_ + *G*_f0_ + *G*_b0_)∕2 and $c=\sqrt{b^{2}-\left(G_{\text{d}1}G_{\text{d}2}+G_{\text{d}1}G_{\text{b}0}+G_{\text{d}2}G_{\text{f}0}\right)}$. Two useful relationships follow from the previous equations:

(A6) $$\begin{array}{rcl} \Lambda_{1}+\Lambda_{2} & = & G_{\text{d}1}+G_{\text{d}2}+G_{\text{f}0}+G_{\text{b}0} \end{array}$$

(A7) $$\begin{array}{rcl} \Lambda_{1}\cdot \Lambda_{2} & = & G_{\text{d}1}G_{\text{d}2}+G_{\text{d}1}G_{\text{b}0}+G_{\text{d}2}G_{\text{f}0} \end{array}$$

The current after the light is turned off is given by:

(A8) $$\begin{array}{l} I_{\text{off}}=V(g_{1}\;\cdot \;O_{1\text{off}}+g_{2}\;\cdot \;O_{2\text{off}})\\ \;=I_{\text{slow}}\exp(-\Lambda_{1}t)\;+I_{\text{fast}}\exp(-\Lambda_{2}t), \end{array}$$

where the expression for *I*_slow_ (corresponds to Λ_1_) and *I*_fast_ (corresponds to Λ_2_) components of the off-current can be calculated from the equation above and are given in Nikolic et al. (2009). Note also that *I*_slow_ + *I*_fast_ = *I*_ss_ which is included as an additional constraint in the fitting procedure.

#### Six-state opsin activation rate fitting (step 1b.)

From the differential equations describing the dynamics of a short pulse (Table 2, three-state model):

$$\frac{dO}{dt}=G_{a}\left(t\right)C-G_{d}O$$

where *G*_*a*_(*t*) is given in Nikolic et al., 2007, Appendix 1. If the light illumination occurs from *t* = 0 to *t* = *t*_pulse_, then after the light goes off (*t* > *t*_*pulse*_):

(A9) $$\begin{array}{l} G_{a}\left(t\right)=G_{a}\left[e^{t_{pulse}∕\tau_{opsin}}-1\right]\cdot e^{-t∕\tau_{opsin}}\;, \end{array}$$

where τ_*opsin*_(= 1∕*G*_*o*_) is the time constant of the opsin complex activation (or conformal change) after the retinal isomerization (which happens very quickly upon photon absorption). Now if we use a very short pulse then *t*_*pulse*_ ≪ τ_*opsin*_ so that *C* ≈ 1 (and secondary cycles are not significantly activated), we may apply the standard approximation for small exponents *e*^*x*^ ≈ 1 + *x*:

$$G_{a}\left(t\right)\approx G_{a}\frac{t_{pulse}}{\tau_{opsin}}\cdot e^{-t∕\tau_{opsin}}=Q\cdot e^{-\frac{t}{\tau_{opsin}}}\;,\text{ where }Q=G_{a}\frac{t_{pulse}}{\tau_{opsin}}$$

and so

(A10) $$\begin{array}{l} \frac{dO}{dt}\approx Q\cdot e^{-G_{o}t}-G_{d}O\;. \end{array}$$

The solution of Equation (A10) is the limit case for very short pulses (relative to τ_*opsin*_) when *I*(*t*) stops being dependent on the pulse duration *t*_*pulse*_:

(A11) $$\begin{array}{l} I\left(t\right)=g\cdot \left(v-E\right)\cdot \frac{Q}{\left(G_{o}-G_{d}\right)}\cdot e^{-t∕\tau_{d}}\cdot \left(1-e^{-\left(G_{o}-G_{d}\right)t}\right)\;. \end{array}$$

Differentiating Equation (A11) we find the time of the peak current:

$$t_{maxlag}=\frac{\log\left(G_{o}∕G_{d}\right)}{G_{o}-G_{d}}=\frac{\tau_{opsin}\tau_{d}}{\tau_{d}-\tau_{opsin}}\log\left(\frac{\tau_{d}}{\tau_{opsin}}\right)\;,$$

where τ_*d*_ = 1∕*G*_*d*_. Assuming that τ_*opsin*_ ≪ τ_*d*_ then,

(A12) $$\begin{array}{l} t_{maxlag}\approx \tau_{opsin}\log\left(\frac{\tau_{d}}{\tau_{opsin}}\right)\;. \end{array}$$

To find *t*_*maxlag*_ we can fit the following function to a series of short pulses:

$$t_{peak}=t_{pulse}+t_{maxlag}\cdot e^{-k\cdot t_{pulse}}\;.$$

Alternatively, estimate *t*_*maxlag*_ from the “delta” protocol as *t*_*pulse*_ → 0. Once we have obtained an estimate for *t*_*maxlag*_ we can then solve for τ_*opsin*_ iteratively as follows:

(A13) $$\begin{array}{l} \tau_{opsin,i+1}=\frac{t_{maxlag}}{\left(t_{maxlag}∕\tau_{d}\right)-\log\left(\tau_{opsin,i}∕\tau_{d}\right)}\;. \end{array}$$

## References

[B1] Adamantidis A. R. Zhang F. Aravanis A. M. Deisseroth K. de Lecea L. (2007). Neural substrates of awakening probed with optogenetic control of hypocretin neurons. Nature 450, 420–424. 10.1038/nature06310 17943086 PMC6744371

[B2] Airan R. D. Thompson K. R. Fenno L. E. Bernstein H. Deisseroth K. (2009). Temporally precise *in vivo* control of intracellular signalling. Nature 458, 1025–1029. 10.1038/nature07926 19295515

[B3] Aravanis A. M. Wang L.-P. Zhang F. Meltzer L. A. Mogri M. Z. Schneider M. B.. (2007). An optical neural interface: *in vivo* control of rodent motor cortex with integrated fiberoptic and optogenetic technology. J. Neural Eng. 4, S143–S156. 10.1088/1741-2560/4/3/s02 17873414

[B4] Arenkiel B. R. Peca J. Davison I. G. Feliciano C. Deisseroth K. Augustine G. J.. (2007). *In vivo* light-induced activation of neural circuitry in transgenic mice expressing channelrhodopsin-2. Neuron 54, 205–218. 10.1016/j.neuron.2007.03.005 17442243 PMC3634585

[B5] Arlow R. L. Foutz T. J. McIntyre C. C. (2013). Theoretical principles underlying optical stimulation of myelinated axons expressing channelrhodopsin-2. Neuroscience 248, 541–551. 10.1016/j.neuroscience.2013.06.031 23811392 PMC4116477

[B6] Arrenberg A. B. Stainier D. Y. R. Baier H. Huisken J. (2010). Optogenetic control of cardiac function. Science 330, 971–974. 10.1126/science.1195929 21071670

[B7] Avants B. W. Murphy D. B. Dapello J. A. Robinson J. T. (2015). Neuropg: open source software for optical pattern generation and data acquisition. Front. Neuroeng. 8:1 10.3389/fneng.2015.00001 PMC4345891 25784873

[B8] Ayling O. G. S. Harrison T. C. Boyd J. D. Goroshkov A. Murphy T. H. (2009). Automated light-based mapping of motor cortex by photoactivation of channelrhodopsin-2 transgenic mice. Nat. Methods 6, 219–224. 10.1038/nmeth.1303 19219033

[B9] AzimiHashemi N. Erbguth K. Vogt A. Riemensperger T. Rauch E. Woodmansee D.. (2014). Synthetic retinal analogues modify the spectral and kinetic characteristics of microbial rhodopsin optogenetic tools. Nat. Commun. 5, 1–12. 10.1038/ncomms6810 25503804

[B10] Bamann C. Kirsch T. Nagel G. Bamberg E. (2008). Spectral characteristics of the photocycle of channelrhodopsin-2 and its implication for channel function. J. Mol. Biol. 375, 686–694. 10.1016/j.jmb.2007.10.072 18037436

[B11] Bamberg E. Bamann C. Feldbauer K. Kleinlogel S. Spitz J. Zimmermann D. (2008). Channelrhodopsins: Molecular Properties and Applications. Washington, DC: Society for Neuroscience.

[B12] Berndt A. Yizhar O. Gunaydin L. A. Hegemann P. Deisseroth K. (2008). Bi-stable neural state switches. Nat. Neurosci. 12, 229–234. 10.1038/nn.2247 19079251

[B13] Boyden E. S. Zhang F. Bamberg E. Nagel G. Deisseroth K. (2005). Millisecond-timescale, genetically targeted optical control of neural activity. Nat. Neurosci. 8, 1263–1268. 10.1038/nn1525 16116447

[B14] Boyle P. M. Williams J. C. Ambrosi C. M. Entcheva E. Trayanova N. A. (2013). A comprehensive multiscale framework for simulating optogenetics in the heart. Nat. Commun. 4, 1–9. 10.1038/ncomms3370 23982300 PMC3838435

[B15] Bruegmann T. Sasse P. (2015). Optogenetic cardiac pacemakers: science or fiction? Trends Cardiovas. Med. 25, 82–83. 10.1016/j.tcm.2014.10.016 25467240

[B16] Busskamp V. Roska B. (2011). Optogenetic approaches to restoring visual function in retinitis pigmentosa. Curr. Opin. Neurobiol. 21, 1–5. 10.1016/j.conb.2011.06.001 21708457

[B17] Chow B. Y. Han X. Dobry A. S. Qian X. Chuong A. S. Li M.. (2010). High-performance genetically targetable optical neural silencing by light-driven proton pumps. Nature 463, 98–102. 10.1038/nature08652 20054397 PMC2939492

[B18] Chuong A. S. Miri M. L. Busskamp V. Matthews G. A. C. Acker L. C. Sørensen A. T.. (2014). Noninvasive optical inhibition with a red-shifted microbial rhodopsin. Nat. Neurosci. 17, 1123–1129. 10.1038/nn.3752 24997763 PMC4184214

[B19] Davison A. P. Brüderle D. Eppler J. M. Kremkow J. Muller E. Pecevski D. (2009). Pynn: a common interface for neuronal network simulators. Front. Neuroinform. 2:11 10.3389/neuro.11.011.2008 19194529 PMC2634533

[B20] Degenaar P. Grossman N. Memon M. A. Burrone J. Dawson M. Drakakis E.. (2009). Optobionic vision: a new genetically enhanced light on retinal prosthesis. J. Neural Eng. 6:035007. 10.1088/1741-2560/6/3/035007 19458396

[B21] Eppler J. M. Helias M. Muller E. Diesmann M. Gewaltig M.-O. (2008). Pynest: A convenient interface to the nest simulator. Front. Neuroinform. 2:12 10.3389/neuro.11.012.2008 19198667 PMC2636900

[B22] Ernst O. P. Sánchez Murcia P. A. Daldrop P. Tsunoda S. P. Kateriya S. Hegemann P. (2008). Photoactivation of channelrhodopsin. J. Biol. Chem. 283, 1637–1643. 10.1074/jbc.M708039200 17993465

[B23] Feldbauer K. Zimmermann D. Pintschovius V. Spitz J. Bamann C. Bamberg E. (2009). Channelrhodopsin-2 is a leaky proton pump. Proc. Natl. Acad. Sci. U.S.A. 106, 12317–12322. 10.1073/pnas.0905852106 19590013 PMC2718366

[B24] Foutz T. J. Arlow R. L. McIntyre C. C. (2012). Theoretical principles underlying optical stimulation of a channelrhodopsin-2 positive pyramidal neuron. J. Neurophysiol. 107, 3235–3245. 10.1152/jn.00501.2011 22442566 PMC3378402

[B25] Gleeson P. Crook S. Cannon R. C. Hines M. L. Billings G. O. Farinella M. (2010). Neuroml: a language for describing data driven models of neurons and networks with a high degree of biological detail. PLoS Comput. Biol. 6:e1000815 10.1371/journal.pcbi.1000815 20585541 PMC2887454

[B26] Goodman D. Brette R. (2008). Brian: a simulator for spiking neural networks in python. Front. Neuroinform. 2:5. 10.3389/neuro.11.005.2008 19115011 PMC2605403

[B27] Goodman D. F. M. Brette R. (2009). The brian simulator. Front. Neurosci. 3, 192–197. 10.3389/neuro.01.026.2009 20011141 PMC2751620

[B28] Gorchetchnikov A. Cannon R. Clewley R. Cornelis H. Davison A. De Schutter E. (2011). Nine*ML*: declarative, mathematically-explicit descriptions of spiking neuronal networks. Front. Neuroinform. Conference Abstract: 4th INCF Congress of Neuroinformatics. 10.3389/conf.fninf.2011.08.00098

[B29] Gradinaru V. Mogri M. Thompson K. R. Henderson J. M. Deisseroth K. (2009). Optical deconstruction of parkinsonian neural circuitry. Science 324, 354–359. 10.1126/science.1167093 19299587 PMC6744370

[B30] Gradinaru V. Thompson K. R. Zhang F. Mogri M. Kay K. Schneider M. B.. (2007). Targeting and readout strategies for fast optical neural control *in vitro* and *in vivo*. J. Neurosci. 27, 14231–14238. 10.1523/JNEUROSCI.3578-07.2007 18160630 PMC6673457

[B31] Gradmann D. Berndt A. Schneider F. Hegemann P. (2011). Rectification of the channelrhodopsin early conductance. Biophys. J. 101, 1057–1068. 10.1016/j.bpj.2011.07.040 21889442 PMC3325117

[B32] Gradmann D. Ehlenbeck S. Hegemann P. (2002). Modeling light-induced currents in the eye of chlamydomonas reinhardtii. J. Mem. Biol. 189, 93–104. 10.1007/s00232-002-1006-8 12235485

[B33] Grossman N. Nikolic K. Toumazou C. Degenaar P. (2011). Modeling study of the light stimulation of a neuron cell with channelrhodopsin-2 mutants. IEEE Trans. Biomed. Eng. 58, 1742–1751. 10.1109/TBME.2011.2114883 21324771

[B34] Grossman N. Simiaki V. Martinet C. Toumazou C. Schultz S. R. Nikolic K. (2013). The spatial pattern of light determines the kinetics and modulates backpropagation of optogenetic action potentials. J. Comp. Neurosci. 34, 477–488. 10.1007/s10827-012-0431-7 23179855 PMC3650242

[B35] Gunaydin L. A. Yizhar O. Berndt A. Sohal V. S. Deisseroth K. Hegemann P. (2010). Ultrafast optogenetic control. Nat. Neurosci. 13, 387–392. 10.1038/nn.2495 20081849

[B36] Han X. Boyden E. S. (2007). Multiple-color optical activation, silencing, and desynchronization of neural activity, with single-spike temporal resolution. PLoS ONE 2:e299. 10.1371/journal.pone.0000299 17375185 PMC1808431

[B37] Hegemann P. Ehlenbeck S. Gradmann D. (2005). Multiple photocycles of channelrhodopsin. Biophys. J. 89, 3911–3918. 10.1529/biophysj.105.069716 16169986 PMC1366958

[B38] Hegemann P. Möglich A. (2011). Channelrhodopsin engineering and exploration of new optogenetic tools. Nat. Methods 8, 39–42. 10.1038/nmeth.f.327 21191371

[B39] Hernandez V. H. Gehrt A. Reuter K. Jing Z. Jeschke M. Mendoza Schulz A.. (2014). Optogenetic stimulation of the auditory pathway. J. Clin. Invest. 124, 1114–1129. 10.1172/JCI69050 24509078 PMC3934189

[B40] Hines M. Davison A. P. Muller E. (2009). Neuron and python. Front. Neuroinform. 3:1. 10.3389/neuro.11.001.2009 19198661 PMC2636686

[B41] Hines M. L. Carnevale N. T. (2000). Expanding neuron's repertoire of mechanisms with nmodl. Neural Comput. 12, 995–1007. 10.1162/089976600300015475 10905805

[B42] Ishizuka T. Kakuda M. Araki R. Yawo H. (2006). Kinetic evaluation of photosensitivity in genetically engineered neurons expressing green algae light-gated channels. Neurosci. Res. 54, 85–94. 10.1016/j.neures.2005.10.009 16298005

[B43] Klapoetke N. C. Murata Y. Kim S. S. Pulver S. R. Birdsey-Benson A. Cho Y. K.. (2014). Independent optical excitation of distinct neural populations. Nat. Meth. 11, 338–346. 10.1038/nmeth.2836 24509633 PMC3943671

[B44] Konermann S. Brigham M. D. Trevino A. E. Hsu P. D. Heidenreich M. Le C.. (2013). Optical control of mammalian endogenous transcription and epigenetic states. Nature 500, 472–476. 10.1038/nature12466 23877069 PMC3856241

[B45] Kuhne J. Eisenhauer K. Ritter E. Hegemann P. Gerwert K. Bartl F. (2014). Early formation of the ion-conducting pore in channelrhodopsin-2. Angewandte Chemie 54, 4953–4957. 10.1002/anie.201410180 25537168

[B46] Lagali P. S. Balya D. Awatramani G. B. Münch T. A. Kim D. S. Busskamp V.. (2008). Light-activated channels targeted to on bipolar cells restore visual function in retinal degeneration. Nat. Neurosci. 11, 667–675. 10.1038/nn.2117 18432197

[B47] Lin J. Y. (2011). A user's guide to channelrhodopsin variants: features, limitations and future developments. Exp. Physiol. 96, 19–25. 10.1113/expphysiol.2009.051961 20621963 PMC2995811

[B48] Lin J. Y. Lin M. Z. Steinbach P. Tsien R. Y. (2009). Characterization of engineered channelrhodopsin variants with improved properties and kinetics. Biophys. J. 96, 1803–1814. 10.1016/j.bpj.2008.11.034 19254539 PMC2717302

[B49] Liu Y. Jacques S. L. Azimipour M. Rogers J. D. Pashaie R. Eliceiri K. W. (2015). Optogensim: a 3d monte carlo simulation platform for light delivery design in optogenetics. Biomed. Opt. Exp. 6, 4859–4870. 10.1364/BOE.6.004859 26713200 PMC4679260

[B50] Muller E. Bednar J. A. Diesmann M. Gewaltig M.-O. Hines M. Davison A. P. (2015). Python in neuroscience. Front. Neuroinform. 9:11. 10.3389/fninf.2015.00011 25926788 PMC4396193

[B51] Nagel G. Szellas T. Huhn W. Kateriya S. Adeishvili N. Berthold P.. (2003). Channelrhodopsin-2, a directly light-gated cation-selective membrane channel. Proc. Natl. Acad. Sci. U.S.A. 100, 13940–13945. 10.1073/pnas.1936192100 14615590 PMC283525

[B52] Newville M. Stensitzki T. Allen D. B. Ingargiola A. (2014). LMFIT: Non-Linear Least-Square Minimization and Curve-Fitting for Python. Zenodo. 10.5281/zenodo.11813

[B53] Nikolic K. Grossman N. Grubb M. S. Burrone J. Toumazou C. Degenaar P. (2009). Photocycles of channelrhodopsin-2. Photochem. Photobiol. 85, 400–411. 10.1111/j.1751-1097.2008.00460.x 19161406

[B54] Nikolic K. Grossman N. Yan H. Drakakis E. Toumazou C. Degenaar P. (2007). A non-invasive retinal prosthesis - testing the concept, in Engineering in Medicine and Biology Society, EMBS 2007. 29th Annual International Conference of the IEEE (Lyon), 6364–6367. 10.1109/IEMBS.2007.4353811 18003477

[B55] Pérez F. Granger B. E. (2007). IPython: a system for interactive scientific computing. Comput. Sci. Eng. 9, 21–29. 10.1109/MCSE.2007.53

[B56] Petreanu L. Mao T. Sternson S. M. Svoboda K. (2009). The subcellular organization of neocortical excitatory connections. Nature 457, 1142–1145. 10.1038/nature07709 19151697 PMC2745650

[B57] Shoham S. Deisseroth K. (2010). Special issue on optical neural engineering: advances in optical stimulation technology. J. Neural Eng. 7:040201. 10.1088/1741-2560/7/4/040201 20644243

[B58] Stehfest K. Hegemann P. (2010). Evolution of the channelrhodopsin photocycle model. Chemphyschem 11, 1120–1126. 10.1002/cphc.200900980 20349494

[B59] Topalidou M. Leblois A. Boraud T. Rougier N. P. (2015). A long journey into reproducible computational neuroscience. Front. Comput. Neurosci. 9:30. 10.3389/fncom.2015.00030 25798104 PMC4350388

[B60] Wang H. Peca J. Matsuzaki M. Matsuzaki K. Noguchi J. Qiu L.. (2007). High-speed mapping of synaptic connectivity using photostimulation in channelrhodopsin-2 transgenic mice. Proc. Natl. Acad. Sci. U.S.A. 104, 8143–8148. 10.1073/pnas.0700384104 17483470 PMC1876585

[B61] Williams J. C. Xu J. Lu Z. Klimas A. Chen X. Ambrosi C. M.. (2013). Computational optogenetics: empirically-derived voltage- and light-sensitive channelrhodopsin-2 model. PLoS Comput. Biol. 9:e1003220. 10.1371/journal.pcbi.1003220 24068903 PMC3772068

[B62] Yizhar O. Fenno L.E. Davidson T. J, Mogri, M. Deisseroth K. (2011). Optogenetics in neural systems. Neuron 71, 9–34. 10.1016/j.neuron.2011.06.004 21745635

[B63] Zhang F. Vierock J. Yizhar O. Fenno L. E. Tsunoda S. Kianianmomeni A.. (2011). The microbial opsin family of optogenetic tools. Cell 147, 1446–1457. 10.1016/j.cell.2011.12.004 22196724 PMC4166436

[B64] Zhang F. Wang L. P. Boyden E. S. Deisseroth K. (2006). Channelrhodopsin-2 and optical control of excitable cells. Nat. Methods 3, 785–792. 10.1038/nmeth936 16990810

[B65] Zhang F. Wang L. P. Brauner M. Liewald J. F. Kay K. Watzke N.. (2007). Multimodal fast optical interrogation of neural circuitry. Nature 446, 633–639. 10.1038/nature05744 17410168

